# A tunable pair electrochemical strategy for the synthesis of new benzenesulfonamide derivatives

**DOI:** 10.1038/s41598-019-38544-4

**Published:** 2019-03-14

**Authors:** Banafsheh Mokhtari, Davood Nematollahi, Hamid Salehzadeh

**Affiliations:** 1 Faculty of Chemistry, Bu-Ali Sina University, Hamedan, 65178-38683 Iran; 2 Faculty of Chemistry, Kharazmi University, Tehran, 15719-14911 Iran

## Abstract

A green, facile and tunable pair electrochemical process was developed for the synthesis of new benzenesulfonamide derivatives by using reductive controlled potential electrolysis of dinitrobenzene (DNB) in the presence of arylsulfinic acids (ASAs). In addition to the usual features associated with paired electrochemical methods, eg high energy efficient, this method has a tunable characteristic, so that, by adjusting the potential, different products can be synthesized. By applying the potential of −0.4 V vs. Ag/AgCl, *N*-hydroxy-*N*-(4-nitrophenyl)benzenesulfonamide derivatives are selectively formed, while, by applying the potential of −1.1 V vs. Ag/AgCl, the final products are *N*-(4-amino-3-(phenylsulfonyl)phenyl) benzenesulfonamide derivatives. This work beautifully shows the potential applications of the electrochemistry as a powerful tool for the synthesis of organic compounds.

## Introduction

Sulfonamides are an important class of organic compounds with antibacterial activity^1^ toward gut infections, *Pneumocystis jirovecii* pneumonia, urinary tract infections, mucous membrane, toxoplasma encephalitis, Kaposi sarcoma herpes virus infection and *isospora* infections in HIV infection^2,3^. These antibiotics also used in the treatment of various types of animal diseases^4^. On the other hand, antibiotic drug resistance is an irrefutable fact that creates a significant problem in public health and is an obstacle to a successful treatment of diseases^5,6^. These findings indicate that the efforts must be directed toward the synthesis of more potent antibiotics. Accordingly, intense drug discovery programs have led to the synthesis of new sulfonamide derivatives^7–42^. The direct synthesis of *N*-arylsulfonamides via the formation of N-S band is the most general approach^7–10^. Although these methods are efficient, they have two major drawbacks which limit their usefulness. Firstly, they use aromatic amines which are carcinogens^11^ and secondly, they lead to impure products. Other important methods for the synthesis of *N*-arylsulfonamide derivatives is the reaction of sulfonamides as nucleophiles with some organic compounds such as halides^12–15^, alcohols^16–21^, aryl esters^22–24^ and arylboronicacids^25–28^. In these methods although the problem of using aniline has been solved, but some disadvantages such as harsh reaction conditions, tedious workup, using metal catalysts, bases and/or ligands accompany these methods. In another efficient strategy, *N*-arylsulfonamides were synthesized by the reaction of sulfonyl azides or hydrazides with benzoic acids^29^ or arylboronic acids^30^. The main problem of these methods is the use of metal ions such as iridium, copper and palladium which pollute the environment. In this context, we synthesized some new sulfonamide derivatives, via electrochemical oxidation of anilines^31–33^, urazoles^34,35^, nitroso aromatic compounds^36–38^ and 1,2-dihydropyridazine-3,6-dione^39^ in the presence of sulfone derivatives. These methods despite their significant advantages over the traditional methods, have used aniline and similar compounds as starting materials. In other efficient methods, nitroarenes have been used instead of anilines in the reaction with arylsulfonyl hydrazides^40^ and sodium arylsulfinates^41^. Metal catalysts, unsafe solvents, tedious workup and harsh reaction conditions are disadventages associated with these methods.

In this context, we recently reported the synthesis of some new sulfonamide derivatives, using simple nitroarenes and sodium arylsulfinates as starting materials, under green conditions^42^. In this study, to extend the scope of our previous work^42^, we report the synthesis of new benzenesulfonamide derivatives by using the reductive controlled potential electrolysis of dinitrobenzene in the presence of arylsulfinic acids sodium salt. The most important features of this method are safe starting materials, catalyst free condition, safe solvent and easy workup. In addition, the unique features of this method compared to our recent paper^42^, is its tunable nature for the synthesis of products. In this approach, different products can be obtained just by changing the applied potential.

## Results and Discussion

### Electrochemical reduction of *p*-dinitrobenzene (DNB)

**Figure 1:**
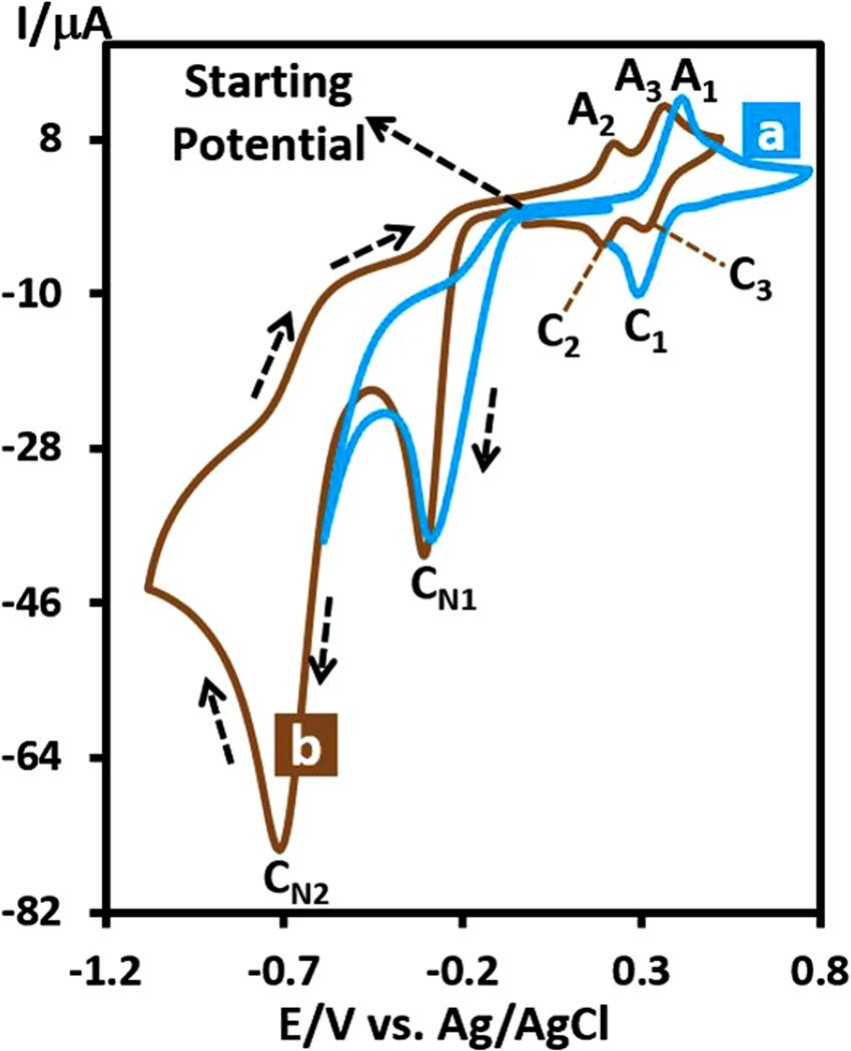
Cyclic voltammograms of 1.0 mM of **DNB** at glassy carbon electrode, in aqueous solution containing phosphate buffer (*c* = 0.2 M, pH = 3.5). Scan rate: 100 mV s^−1^. Temperature: 25 ± 1 °C. (**a**) Cathodic switching potential, −0.60 V and (**b**) cathodic switching potential −1.1 V.

**Figure 2:**
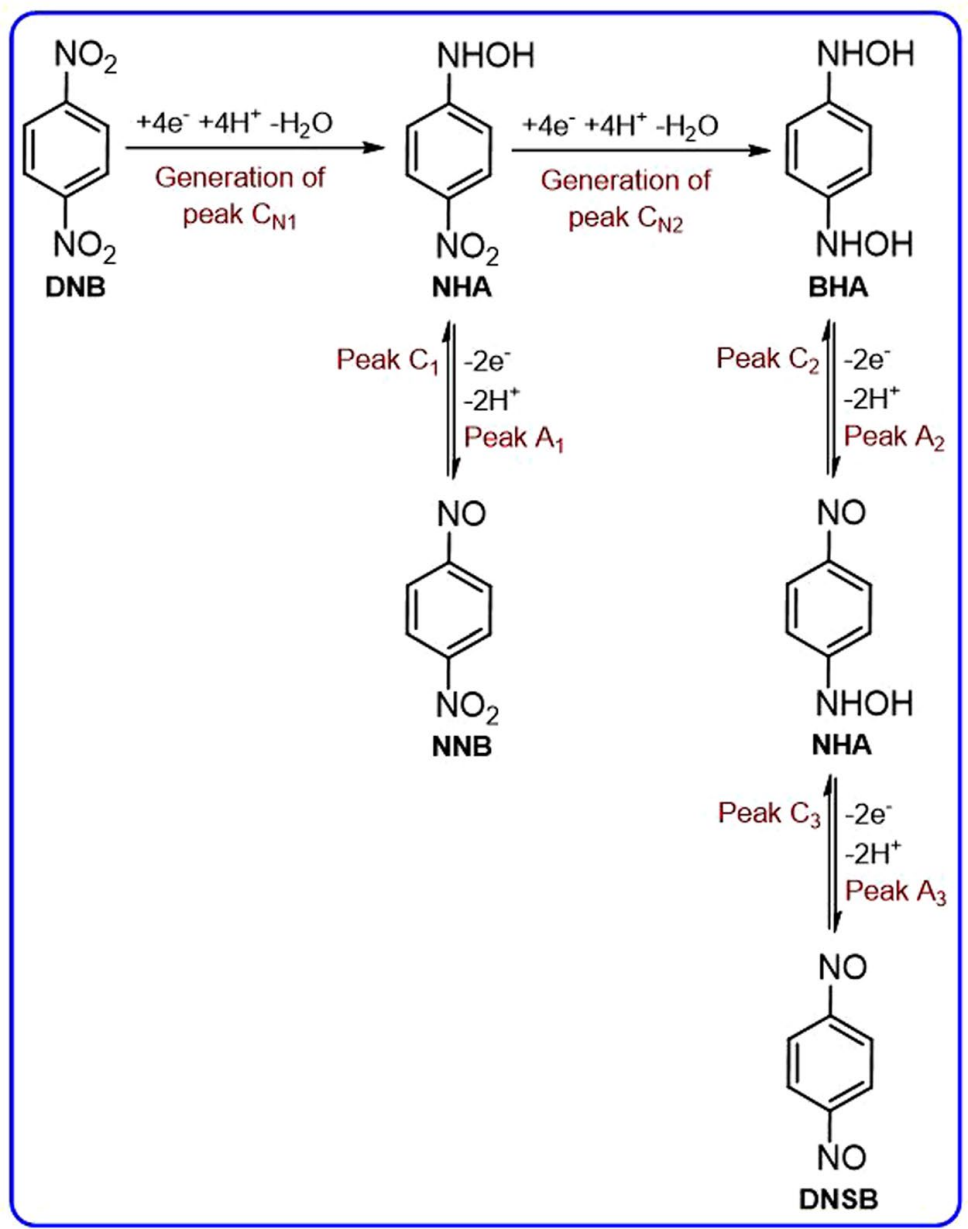
Proposed mechanism for the electrochemical behavior of **DNB**.

We report here the electrochemical behavior of **DNB**. The cyclic voltammograms of **DNB** (1.0 mM) at glassy carbon electrode, in water solution containing phosphate buffer (*c* = 0.2 M, pH = 3.5) at scan rate, 100 mV s^−1^ are shown in Fig. 1. The presence of two nitro groups has caused the creation of two cathodic peaks (C_N1_ and C_N2_). The electrochemical behaviour of **DNB** clearly depends on the switching potential and scan potential direction, so when the potential was scanned from 0.00 to + 0.80 V vs. Ag/AgCl and back, no anodic or cathodic peaks were observed in the sweeping area. However, when the potential direction was reversed and scanned in the negative potential direction (−0.60 V), a well-defined irreversible cathodic peak (C_N1_) is observed at −0.29 V/Ag/AgCl, correspond to the reduction of one of the nitro groups of **DNB** to hydroxylamine group. Under these conditions, in the reverse scan, an anodic peak, A_1_ (*E*_pA1_ = 0.42 V), ascribed to the oxidation of *N*-(4-nitrophenyl)hydroxylamine (**NHA**) to 1-nitro-4-nitrosobenzene (**NNB**) and its cathodic counterpart (C_1_) which is correspond to the reduction of **NNB** to **NHA** were observed (Fig. 2)^42,43^.

However, when the switching potential was changed to −1.1 V/Ag/AgCl, both nitro groups reduced to hydroxylamine groups produces *N*,*N*′-(1,4-phenylene)bis(hydroxylamine) (**BHA**). Under these conditions, in the reverse scan, two successive oxidation processes, makes the anodic peaks A_2_ and A_3_. These peaks are related to the oxidation of **BHA** to *N*-(4-nitrosophenyl) hydroxylamine (**NHA**) (peak A_2_) and oxidation of **NHA** to 1,4-dinitrosobenzene (**DNSB**) (peak A_3_), respectively. Obviously, the cathodic peaks C_3_ and C_2_, are related to the reduction of **DNSB** to **NHA** and reduction of **NHA** to **BHA** (Fig. 2).

**Figure 3:**
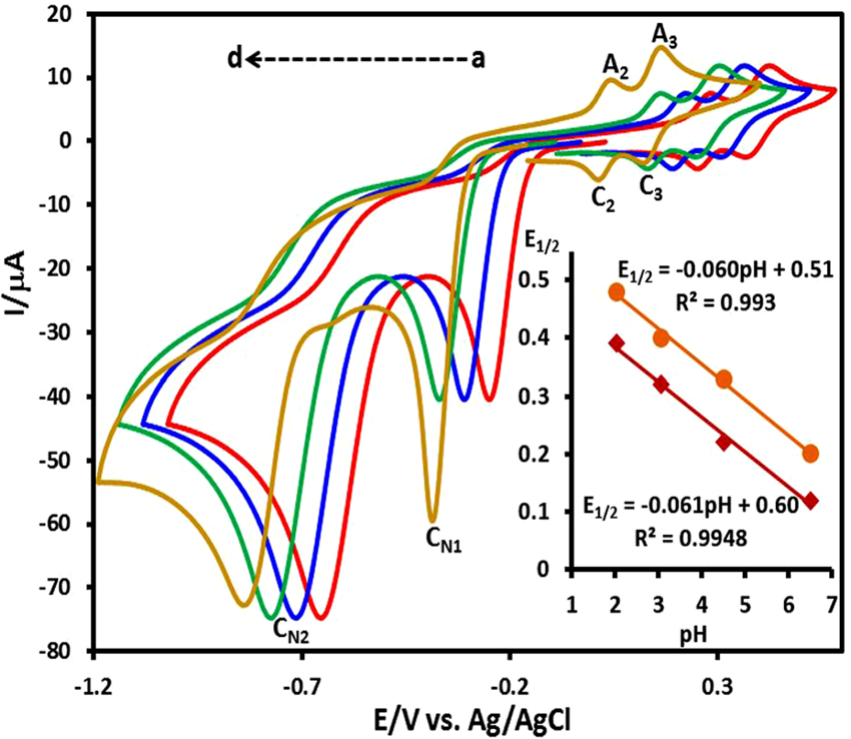
Cyclic voltammograms of 1.0 mM of **DNB** at glassy carbon electrode, in water (with different pH values and same ionic strength)/ethanol (80/20, v/v) mixture. pHs from (**a**) to (**d**) are: 2.0, 3.1, 4.5 and 6.5. Scan rate: 100 mV s^−1^. Temperature: 25 ± 1 °C. Inset: the potential-pH diagram for **BHA**/**NHA** (redox couple A_2_/C_2_) and **NHA**/**DNSB** (redox couple A_3_/C_3_).

In addition, we have found that, the change of potential scan rate has no significant effect on the cyclic voltammograms **DNB** in the range of 10–100 mV s^−1^(see, Supporting Information). The effect of pH on the cyclic voltammogram of **DNB** is shown in Fig. 3. As can be seen, all anodic and cathodic peaks are pH-dependent and shift negatively with the increase of pH. The potential-pH diagrams for **BHA**/**NHA** (redox couple A_2_/C_2_) and **NHA**/**DNSB** (redox couple A_3_/C_3_) are shown in Fig. 3, inset. As can be seen, in the studied pH range (2.0–6.5), both lines have a slope of ∼60 mV which are consistent with the two-electron/two-proton processes.

**Figure 4:**
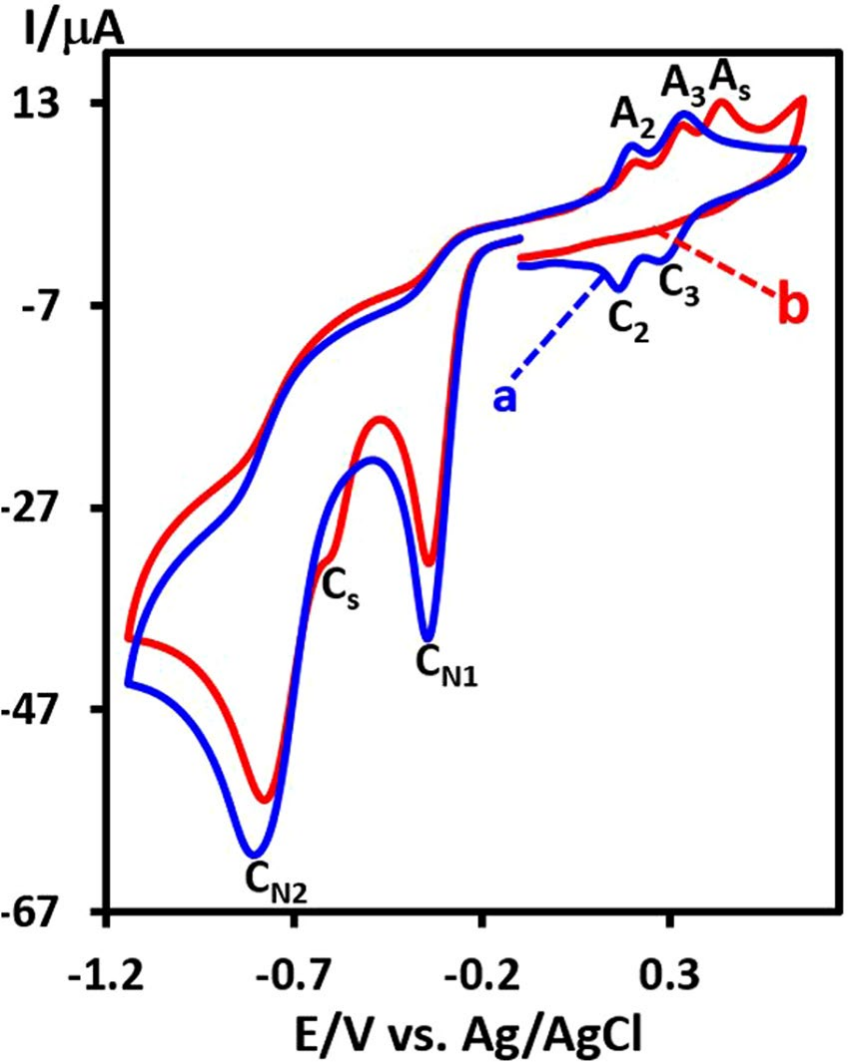
Cyclic voltammograms of 1.0 mM **DNB**: (**a**) in the absence and b) in the presence of **BSA** (1.0 mM) at glassy carbon electrode, in aqueous solution buffer (*c* = 0.2 M, pH = 3.5). Scan rate: 100 mV s^−1^. Temperature: 25 ± 1 °C.

The effect of benzenesulfinic acid (**BSA**) on the cyclic voltammogram of **DNB** is shown in Fig. 4b. The comparison of this voltammogram with the voltammogram of **DNB** in the absence of **BSA** (Fig. 4a), shows three significant changes. (1) The appearance of a new anodic peak (A_s_) at 0.44 V/Ag/AgCl. (2) The disappearance of the cathodic peaks C_2_ and C_3_ and (3) the appearance of a new cathodic peak (C_s_) at −0.60 V/Ag/AgCl. The disappearance of the cathodic peaks C_2_ and C_3_ are evidence that the nitroso compounds **NHA** and **DNSB** are removed from the electrode surface by reaction with **BSA**. In addition, the appearance of the new anodic and cathodic peaks A_s_ and C_s_ confirms the formation of an organic compound with the oxidation potential more than **DNB**.

**Figure 5:**
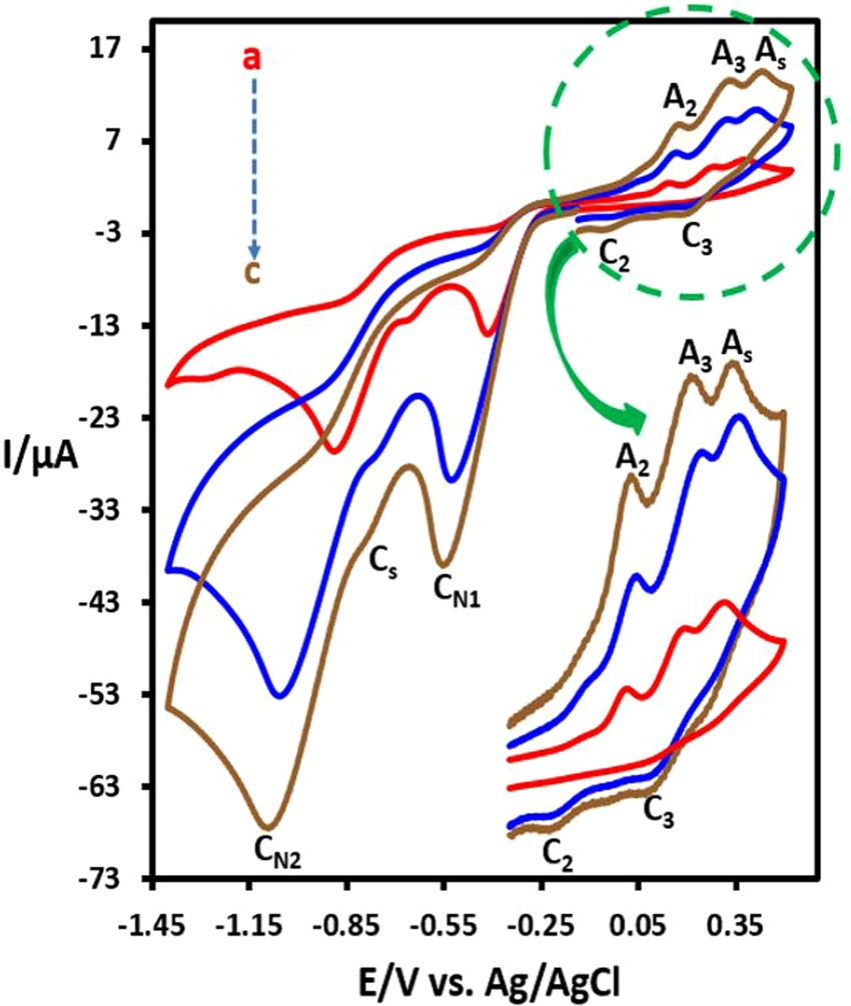
Cyclic voltammograms of 1.0 mM **DNB** in the presence of **BSA** (1.0 mM) at glassy carbon electrode, in aqueous solution buffer (*c* = 0.2 M, pH = 3.5) at different scan rates. Scan rates from a to c are: 10, 50 and 100 mV s^−1^, respectively. Temperature: 25 ± 1 °C.

The effect of potential scan rate on the cyclic voltammograms of **DNB** in the presence of **BSA** is shown in Fig. 5. The significant change is the reappearance of C_2_ and C_3_ peaks at higher potential scan rates. When the potential scan rate increases, the reaction time for the reaction of **BSA** with electrochemically generated **NHA** and **DNSB** decreased so that, the unreacted **NHA** and **DNSB** produce cathodic peaks C_2_ and C_3_ in the reverse scan.

**Figure 6:**
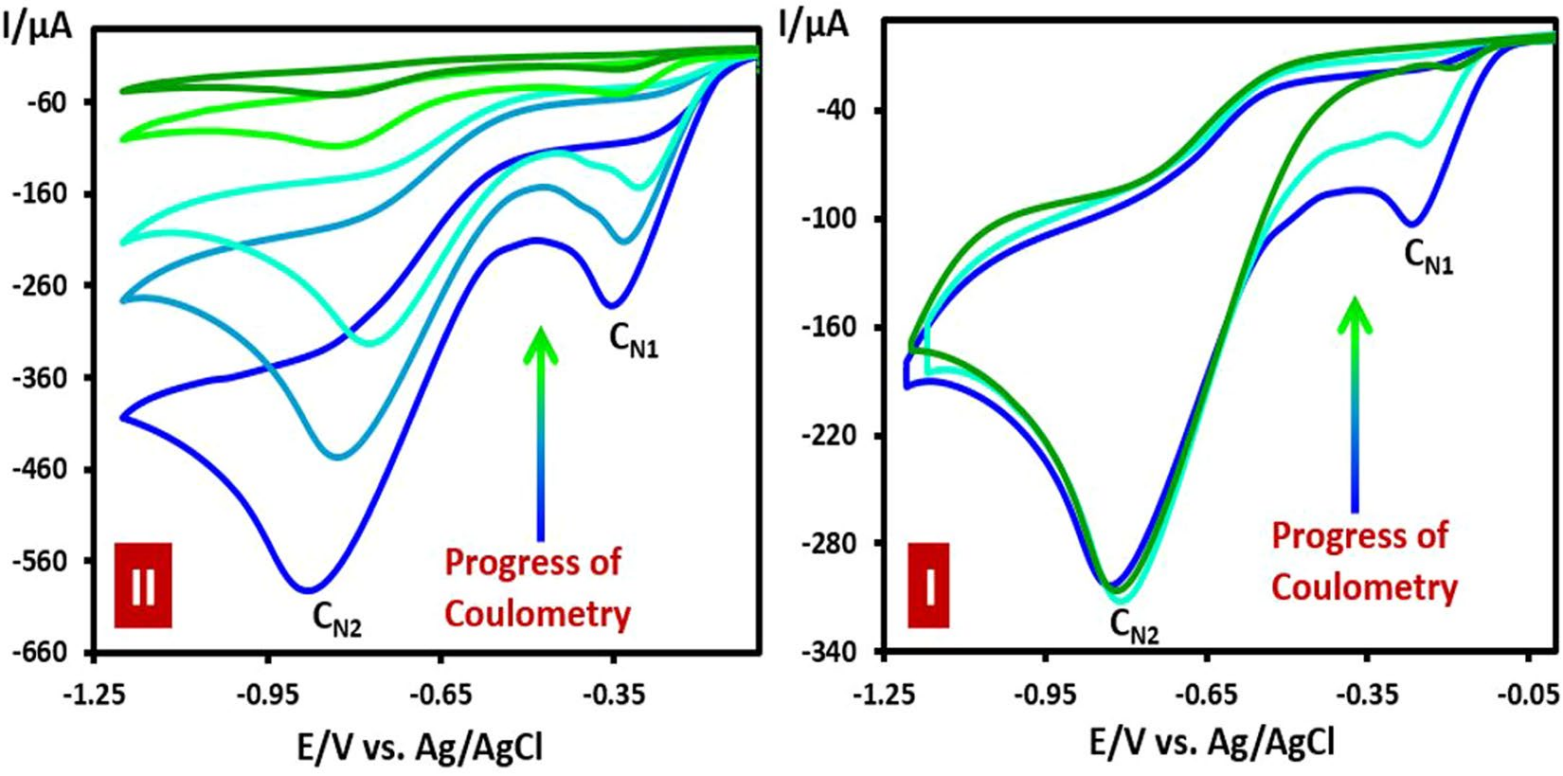
Cyclic voltammograms of **DNB** during controlled-potential coulometry: I) **DNB** (1.0 mmol) in the presence of **BSA** (1.0 mmol) at −0.4. II) **DNB** (2.0 mmol) in the presence of **BSA** (4.0 mmol) at −1.1 V. Working electrode; glassy carbon. Solvent; aqueous solution containing phosphate buffer (*c* = 0.2 M, pH = 3.5). Scan rate: 100 mV s^−1^; Temperature: 25 ± 1 °C.

Since **DNB** have two cathodic peaks, controlled-potential method was used for its selective reduction. Two electrolysis experiments were carried out in the presence of **ASAs**, at cathodic potentials of −0.4 V and −1.1 V vs. Ag/AgCl, respectively and electrolysis progress was monitored by cyclic voltammetry (Fig. 6). At cathodic potential of −0.4 V, the current of cathodic peak C_N1_ (*I*_pCN1_) decreased with the charge passed and finally disappeared when the charge passed was about 4e^−^ per molecule of **DNB**. Under these conditions, *I*_pCN2_ does not change. At cathodic potential of −1.1 V, however, both cathodic peak currents, *I*_pCN1_ and *I*_pCN2_ decreased with the charge passed and disappeared when the charge passed was about 12e^−^ per molecule of **DNB**.

**Figure 7:**
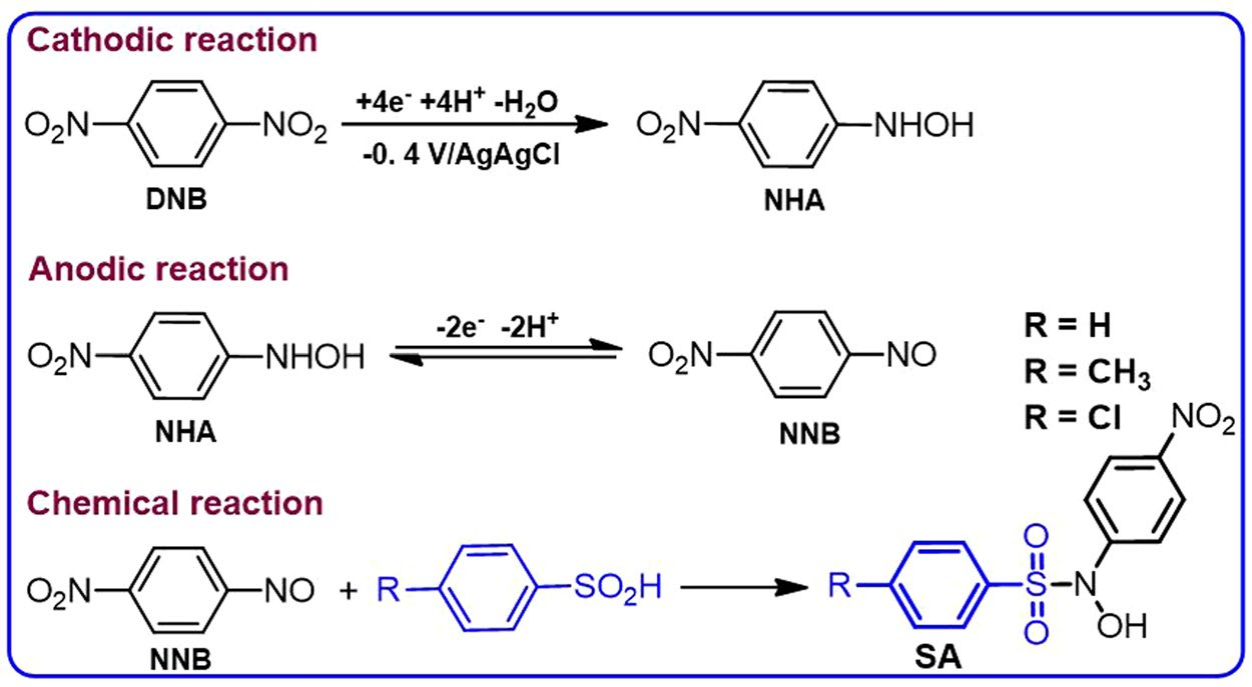
Proposed mechanism for the electrochemical synthesis of **SA** derivatives.

**Figure 8:**
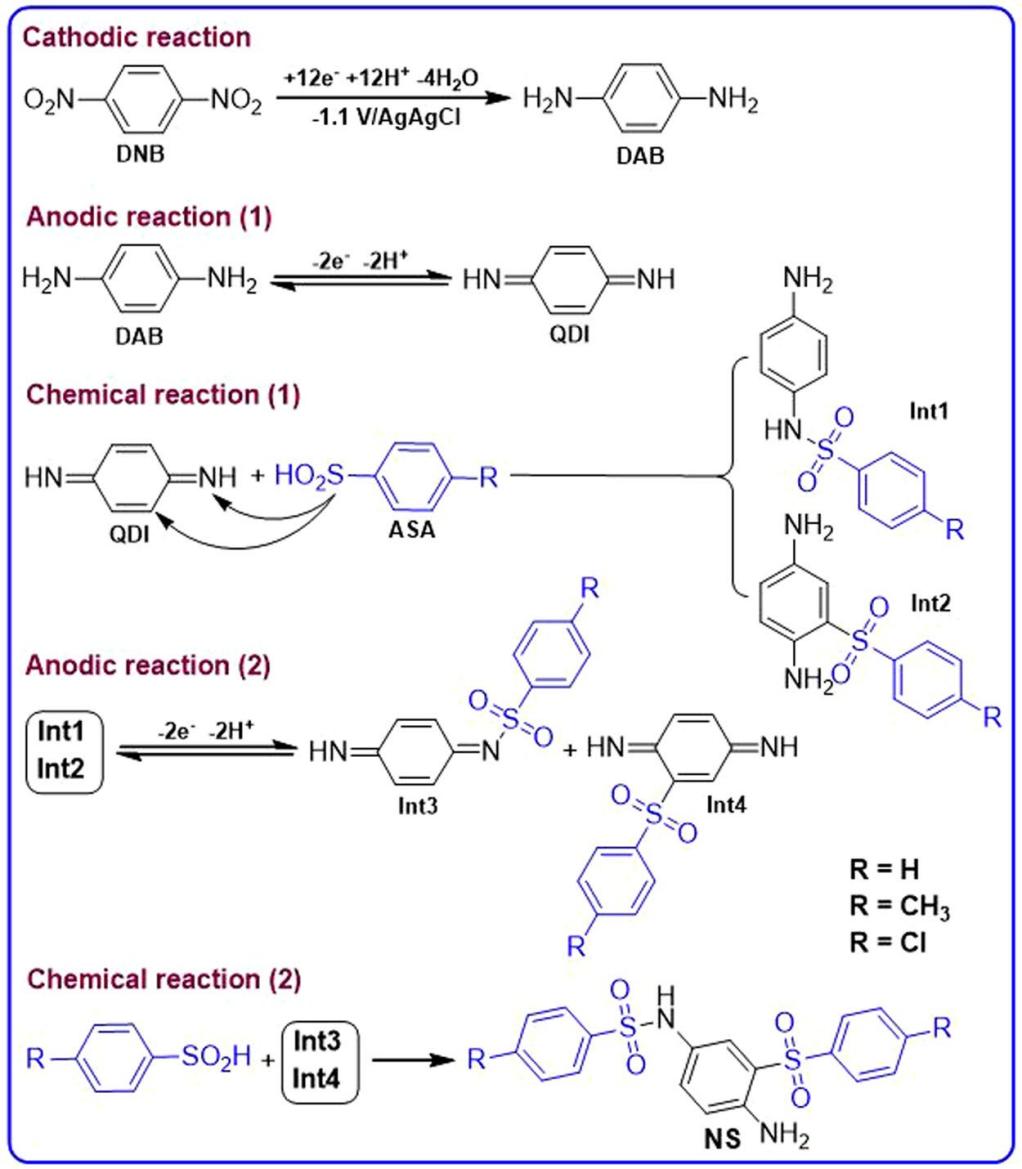
Proposed mechanism for the electrochemical synthesis of **NS** derivatives.

The reaction scheme for the synthesis of benzenesulfonamides **SA** and **DS** is given in Figs 7 and 8. Holding the electrode potential at −0.4 V vs. Ag/AgCl is the essential condition for the synthesis of the benzenesulfonamides **SA1-SA3** (Fig. 7). However, when the electrode potential reached −1.1 V/Ag/AgCl, the main product, benzenesulfonamides **NS1-NS3** are produced after reduction of **DNB** to *p*-diaminobenzene (Fig. 8). As shown in Fig. 7, the cathodically formed **NHA** is oxidized at the anode to 1-nitro-4-nitrosobenzene (**NNB**). The reaction of **NNB** with **ASAs** as a nucleophile produces the benzenesulfonamides **SA1**-**SA3**.

Figure 8, shows the possible mechanistic pathway for the synthesis of **NS** derivatives. When the applied potential is −1.1 V/Ag/AgCl, two nitro groups after consumption of 12e^−^ per molecule of **DNB**, converts to amine groups. In the next stage, the electrochemically generated 1,4-diaminobenzene (**DAB**), at the anode after passing 2e^−^, is converted to *p*-quinonediimine (**QDI**)^44,45^. The reaction of **ASAs** with **QDI**, along with aromatization, leading to sulfonamide **Int1** (or **Int2**)^31^. It should be noted that **QDI** can be attacked by **ASAs** either form C or N atoms to form two types of intermediates (**Int1** or **Int2**). Another oxidation step along with a chemical reaction converts **Int1** (or **Int2**) to *N*-arylsulfonamide derivatives (**NS1-NS3**) as the final products. According to Fig. 8, the anodic peak A_s_ (Fig. 4) is related to the oxidation of **Int1** (or **Int2**) to the corresponding quinonediimine (**Int3**) (or **Int4**). The presence of an electron withdrawing sulfone group in the structure of **Int1** (or **Int2**) makes its oxidation harder than that of **BHA** (peak A_2_) and **NHA** (peak A_3_).

## Conclusions

The results of this work have three important implications. (a) This work has led to the synthesis of new benzenesulfonamide derivatives that may have medicinal applications. (b) This work has used a simple cell and electrodes, safe starting materials, catalyst free condition, safe solvent, easy workup room temperature conditions and low energy consumption (because of pair strategy) for the synthesis of title compounds **SA1**-**SA3** and **NS1-NS3**.(c) This work reports a tunable electrochemical method for the synthesis of **SA1**-**SA3** and **NS1-NS3**. In this method, the applied potential for the synthesis of benzenesulfonamides **SA1**-**SA3**, is −0.4 V, however benzenesulfonamides **NS1-NS3** can be synthesized only by changing the applied potential from −0.4 V to −1.1 V/Ag/AgCl.

## Materials and Methods

### Apparatus and Reagents

All voltammetric and coulometric experiments were done using an Autolab model PGSTAT 20 potentiostat/galvanostat. A glassy carbon disc (1.8 mm diameter) and a platinum wire were used as the working and counter electrodes, respectively. The glassy carbon electrode was polished using alumina slurry followed by washing with water and acetone. The reference electrode used was Ag/AgCl (saturated KCl). All electrodes are prepared from AZAR Electrodes. These electrodes are used in voltammetric experiments. An assembly of two carbon plates (each one, 10 cm lenght, 7 cm width and 1 cm thickness) were used as the anode and cathode in controlled-potential coulometry.

*p*-Dinitrobenzene (**DNB**), arylsulfinic acids sodium salt (**ASAs**), phosphate, acetate salts and ethanol were obtained from commercial sources and were used without further purification. The purity of products has been checked by TLC, and characterization has been done using ^1^H NMR, ^13^C NMR, IR spectroscopic techniques and mass spectrometry.

### Electroorganic synthesis of benzenesulfonamide derivatives

Electroorganic synthesis of **SA** and **NS** derivatives were performed under controlled-potential conditions. In a typical procedure, a solution (80 mL) of water (phosphate buffer, pH = 3.5, *c* = 0.2 M)/ethanol mixture (80/20, v/v) containing *p*-dinitrobenzene (**DNB**) and arylsulfinic acids sodium salt (**ASAs**) was electrolyzed at optimum conditions according (Table 1).

**Table 1:** Experimental conditions used for the synthesis of **SA** and **NS**.

Product	DNB/mmol	ASA/mmol	Applied potential/V	Yield %	mp °C
**SA1**	1.0	1.0	−0.4	76	139–142
**SA2**	1.0	1.0	−0.4	73	137–139
**SA3**	1.0	1.0	−0.4	78	142–143
**NS1**	1.0	2.0	−1.1	60	174–176
**NS2**	1.0	2.0	−1.1	68	172–175
**NS3**	1.0	2.0	−1.1	66	165–168

The electrolysis was terminated when the decay of the current became more than 95%. Since, the products are insoluble in water (phosphate buffer, pH = 3.5, *c* = 0.2 M)/ethanol mixture (80/20, v/v), separation is carried out only by filtration. The collected solids were washed on the filter with distilled water (several times).

**Figure 9:**
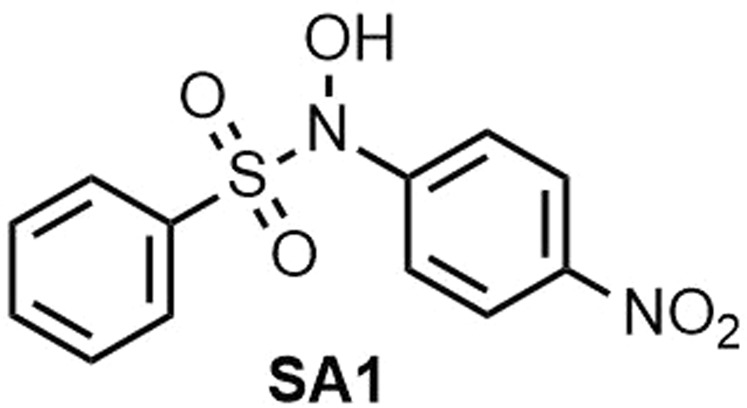
Structure of **SA1**.

***N*-hydroxy-*N*-(4-nitrophenyl)benzenesulfonamide (SA1) (**Fig. 9**)**. Isolated yield: 76%. Mp = 139–142 °C. IR (KBr, cm^−1^): 3336, 3115, 1614, 1591, 1520, 1448, 1348, 1178, 1161, 858, 755, 689, 609. ^1^H NMR, *δ* ppm (400 MHz, acetone-*d*_6_): 7.48 (d, *J* = 11.5 Hz, 2 H), 7.51–7.58 (m, 4H), 7.71–7.74 (m, 1H), 8.18 (d, *J* = 11.0 Hz, 2H), 10.7 (s, 1H, OH). ^13^C NMR, *δ* ppm (100 MHz, acetone-*d*_6_): 122.1, 123.8. 128.8, 129.3, 131.1, 132.9, 134.4, 145.8, 148.4, 148.5. MS (EI) *m/z* (%): 77 (100), 141 (87), 278 (27), 294 [44, (M^+^)].

**Figure 10:**
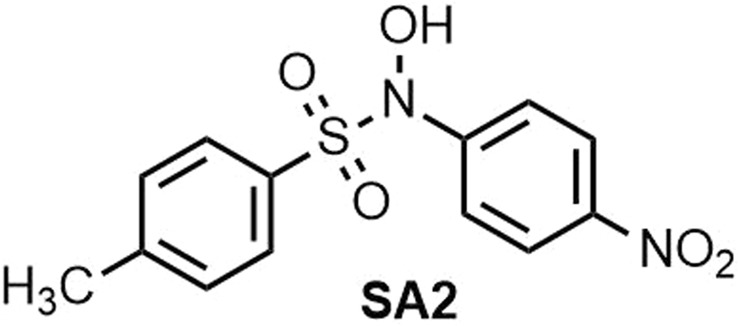
Structure of **SA2**.

***N*-hydroxy-4-methyl-*N*-(4-nitrophenyl)benzenesulfonamide (SA2) (**Fig. 10**)**. Isolated yield: 73%. Mp = 137–139 °C. ^1^H NMR (400 MHz, DMSO-*d*_6_): *δ* ppm 2.37 (s, 3H, CH_3_), 7.37 (d, *J = *3.6 Hz, 3H), 7.44 (t, *J* = 6 Hz, 2H), 7.68–7.71 (m, 1H), 8.20 (d, *J = *9.2 Hz, 2H), 11.49 (d, *J = *3.2 Hz, 1H, OH). ^13^C NMR (100 MHz, DMSO-*d*_6_): *δ* ppm 21.06 CH_3_, 122.0, 124.1, 128.6, 129.0, 129.5, 131.5, 131.7, 145.0, 145.2, 148.3. IR (KBr): 3346, 2959, 2930, 1611, 1522, 1344, 1289, 1166, 857, 669, 556 cm^−1^. MS (EI, 70 eV) *m/z* (relative intensity) 77 (100), 141 (31), 278 (26), 308 [50, (M^+^)].

**Figure 11:**
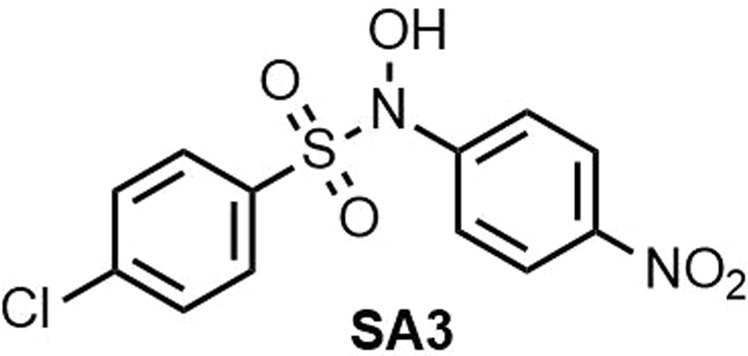
Structure of **SA3**.

**4-Chloro-*N*-hydroxy-*N*-(4-nitrophenyl)benzenesulfonamide (SA3) (**Fig. 11**)**. Isolated yield: 78%. Mp = 142–143 °C. IR (KBr, cm^−1^): 3404, 1609, 1522, 1589, 1522, 1347, 1183, 1169, 762, 588. ^1^H NMR, *δ* ppm (400 MHz, acetone-*d*_6_): 7.50 (d, *J* = 9.2 Hz, 2H), 7.55–7.60 (m, 4H), 8.20 (d, *J* = 9.2 Hz, 2H), 10.86 (s, 1H, OH). ^13^C NMR, *δ* ppm (100 MHz, acetone-*d*_6_): 122.3, 124.0, 128.8, 129.1, 131.0, 131.4, 140.4, 145.9, 148.1, 148.2. MS (EI) *m/z* (%): 75 (100), 149 (65), 279 (5), 328 [30, (M^+^)].

**Figure 12:**
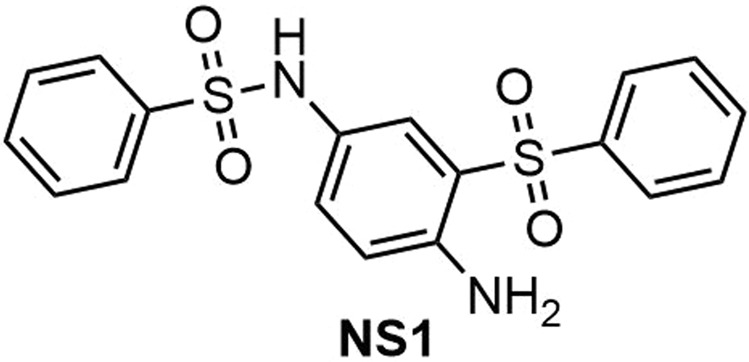
Structure of **NS1**.

***N*-(4-amino-3-(phenylsulfonyl)phenyl)benzenesulfonamide (NS1) (**Fig. 12**)**. Isolated yield: 60%. Mp = 174–176 °C. (Lit. 175–176 °C)^46^. IR (KBr, cm^−1^): 3466, 3360, 1623, 1499, 1466, 1143, 800, 734, 607. ^1^H NMR, *δ* ppm (400 MHz, DMSO-*d*_6_): 4.22 (s, 1H, NH), 5.68 (s, 1H, NH_2_), 6.02 (s, 1H), 6.52 (s, 1H), 6.71 (t, *J* = 16.4 Hz, 1H), 7.19 (s, 1H), 7.35 (s, 1H), 7.68–7.79 (m, 6H), 7.94 (d, *J* = 10.8 Hz 2H). ^13^C NMR, *δ* ppm (125 MHz DMSO-*d*_6_): 117.6, 119.0, 121.0, 125.1, 126.1, 126.6, 128.8, 129.1, 129.5, 130.4, 132.0, 133.9, 139.4, 140.2. MS (EI) *m/z* (%): 77 (100), 110 (80), 149 (86), 170 (20), 279 (6), 387[20, (M^+^ − 1)].

**Figure 13:**
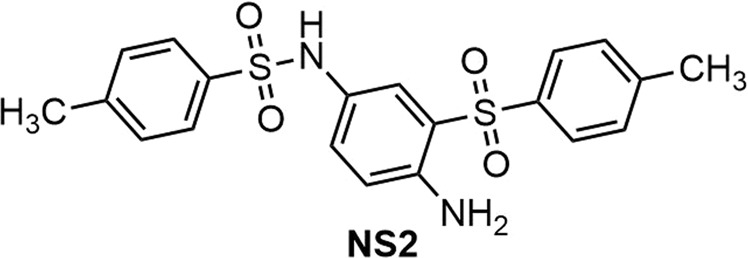
Structure of **NS2**.

***N*-(4-amino-3-tosylphenyl)-4-methylbenzenesulfonamide) (NS2) (**Fig. 13**)**. Isolated yield: 68%. Mp = 172–175 °C.IR (KBr): 3446, 3365, 2958, 1728, 1500, 1285, 1140, 658, 589 cm^−1^. ^1^H NMR (400 MHz, DMSO-*d*_6_): *δ* ppm 2.38 (s, 6H, CH_3_), 4.20 (s, 1H, NH), 5.65 (s, 1H, NH_2_), 6.62 (s, 1H), 6.64–6.81 (m, 2H), 7.11 (d, *J* = 10.4 Hz, 1H), 7.37 (d, *J* = 7.6 Hz, 3H), 7.65–7.85 (m, 4H). ^13^C NMR (100 MHz, acetone-*d*_6_): *δ* ppm 20.52 CH_3_, 113.1, 117.3, 118.1, 118.9, 120.2, 123.1, 124.9, 126.8, 127.7, 128.3, 128.9, 129.2, 129.4, 131.1.MS (EI) *m/z* (%): 77 (100), 141 (27), 278 (15), 418[12, (M^+^ + 2)].

**Figure 14:**
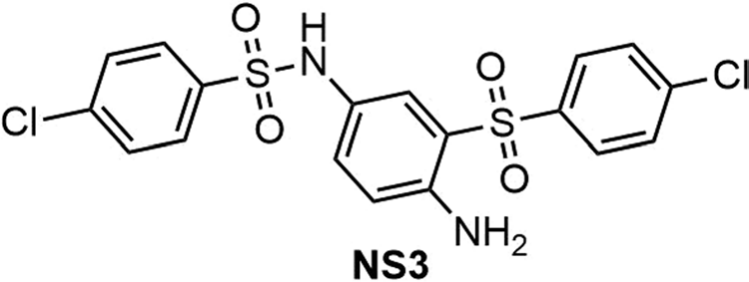
Structure of **NS3**.

***N*-(4-amino-3-(4-chlorophenylsulfonyl)phenyl)-4-chlorobenzenesulfonamide (NS3) (**Fig. 14**)**. Isolated yield: 66%. Mp = 165–168 °C. IR (KBr): 3460, 3376, 1628, 1500, 1315, 1145, 757, 629, 557 cm^−1^. ^1^H NMR (400 MHz, acetone-*d*_6_): *δ* ppm 7.30 (t, *J* = 7.6 Hz, 1H), 7.35–7.40 (m, 3H), 7.45–7.54 (m, 4H), 7.57 (d, *J* = 8.0 Hz, 2H), 7.64 (d, *J* = 8.0 Hz, 2H), 7.76–7.81 (m, 2H). ^13^C NMR (100 MHz, acetone-*d*_6_): *δ* ppm 118.8, 124.3, 126.1, 128.2, 128.3, 128.8, 129.3, 130.6, 131.0, 132.6, 132.7, 134.0, 136.2, 142.1.MS (EI) *m/z* (%): 51 (36), 77 (100), 141 (87), 155 (48), 278 (31), 292(12), 455 [25, (M^+^ – 1)].

## Supplementary information

Supporting Information
