# Supplementary material for: A tunable pair electrochemical strategy for the synthesis of new benzenesulfonamide derivatives
Source: Sci Rep. 2019 Mar 14;9:4537. doi: 10.1038/s41598-019-38544-4 (PMC6418235; doi:10.1038/s41598-019-38544-4)

Supporting Information

A tunable pair electrochemical strategy for the synthesis of new benzenesulfonamide derivatives

Banafsheh Mokhtari[a], Davood Nematollahi*[a] & Hamid Salehzadeh[b]

[a]Faculty of Chemistry, Bu-Ali Sina University, Hamedan, Iran, Zip Code: 65178-38683. E-mail: nemat@basu.ac.ir, Fax:+98-813-8257407

[b]Faculty of Chemistry, kharazmi University, Tehran, Iran, 15719-14911

Contents

1	Cyclic voltammograms of *p*-dinitrobenzene	Page 3
2	Cyclic voltammograms of *p*-dinitrobenzene in the absence and presence of **BSA**	Page 4
3	IR spectrum of **SA1**	Page 5
4	^{1}HNMR Spectrum of **SA1**	Page 6
5	Expanded ^{1}H NMR spectrum of **SA1**	Page 7
6	^{13}C NMR spectrum of **SA1**	Page 8
7	MS spectrum of **SA1**	Page 9
8	IR spectrum of **SA2**	Page 10
9	^{1}HNMR Spectrum of **SA2**	Page 11
10	Expanded ^{1}H NMR spectrum of **SA2**	Page 12
11	^{13}C NMR spectrum of **SA2**	Page 13
12	MS spectrum of **SA2**	Page 14
13	IR spectrum of **SA3**	Page 15
14	^{1}HNMR Spectrum of **SA3**	Page 16
15	Expanded ^{1}H NMR spectrum of **SA3**	Page 17
16	^{13}C NMR spectrum of **SA3**	Page 18
17	MS spectrum of **SA3**	Page 19
18	IR spectrum of **NS1**	Page 20
19	^{1}HNMR Spectrum of **NS1**	Page 21
20	^{13}C NMR spectrum of **NS1**	Page 22
21	^{13}C NMR Expanded spectrum of **NS1**	Page 23
22	MS spectrum of **NS1**	Page 24
23	IR spectrum of **NS2**	Page 25
24	^{1}HNMR Spectrum of **NS2**	Page 26
25	^{13}C NMR spectrum of **NS2**	Page 27
26	MS spectrum of **NS2**	Page 28
27	IR spectrum of **NS3**	Page 29
28	^{1}HNMR Spectrum of **NS3**	Page 30
29	Expanded ^{1}H NMR spectrum of **NS3**	Page 31
30	^{13}C NMR spectrum of **NS3**	Page 32
31	MS spectrum of **NS3**	Page 33

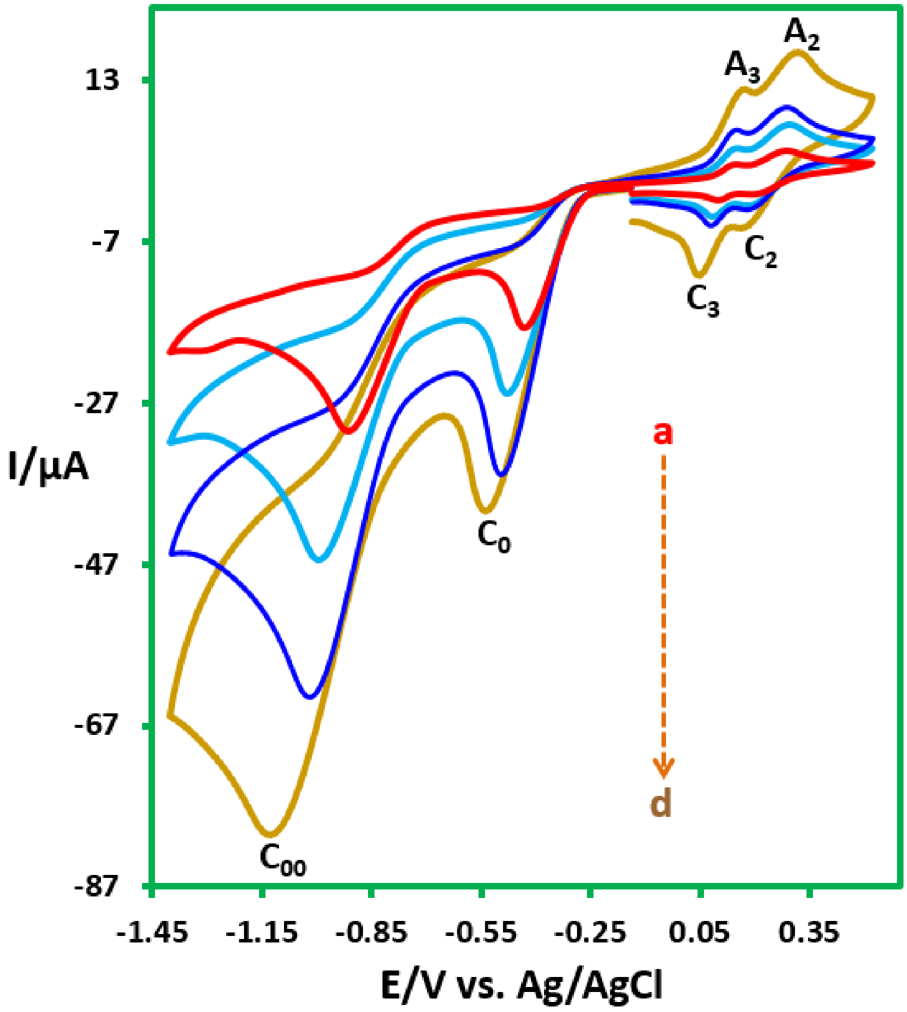

Cyclic voltammograms of 1.0 mM *p*-dinitrobenzene (**DNB**) at glassy carbon electrode, in aqueous solution buffer (c = 0.2 M, pH = 3.5) at different scan rates. Scan rates from a tod are: 10, 25, 50 and 100 mV s^{-1}, respectively. Temperature: 25 ± 1 ^{o}C.

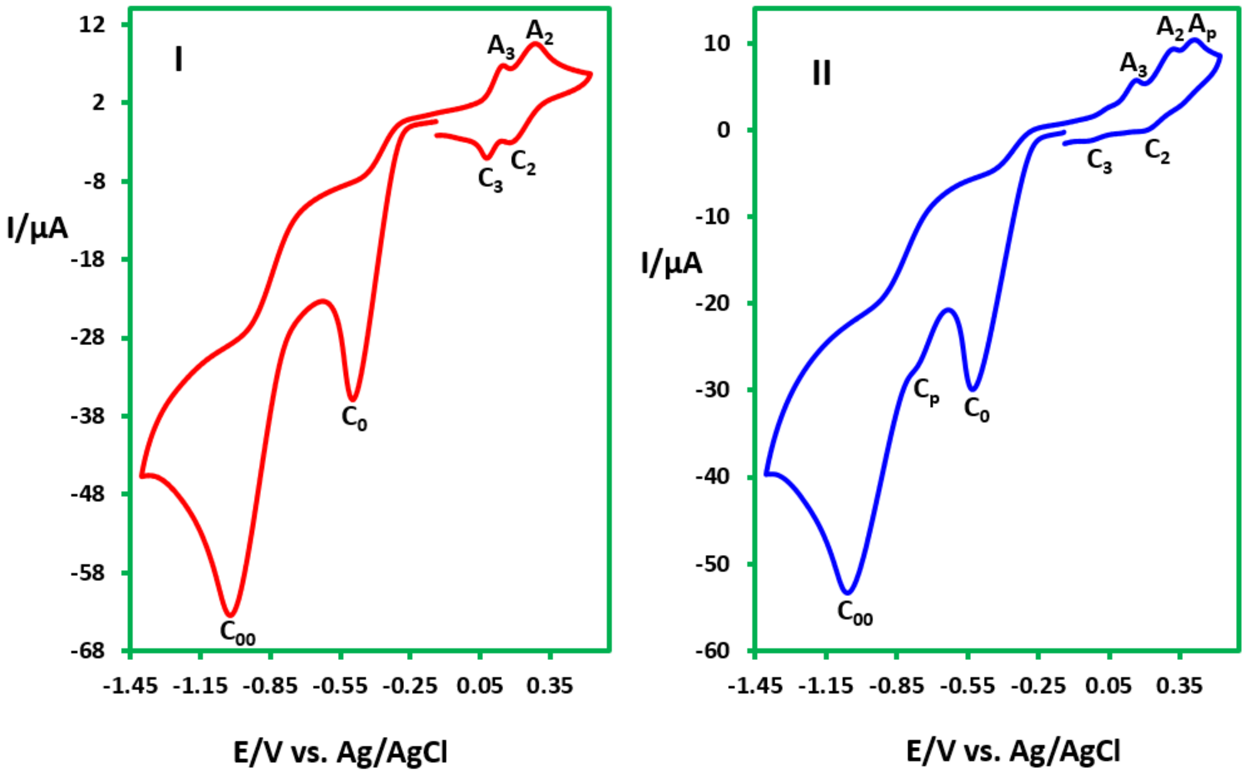

Cyclic voltammograms of 1.0 mM *p*-dinitrobenzene (**DNB**). Part I: in the absence and Part II: in the presence of **BSA** (1.0 mM) at glassy carbon electrode, in aqueous solution buffer (*c* = 0.2 M, pH = 3.5). Scan rate: 50 mV s^{-1}.Temperature: 25 ± 1 ^{o}C.

IR spectrum of SA1

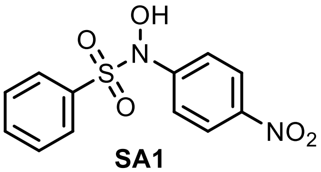

SA1

N-hydroxy-*N*-(4-nitrophenyl)benzenesulfonamide

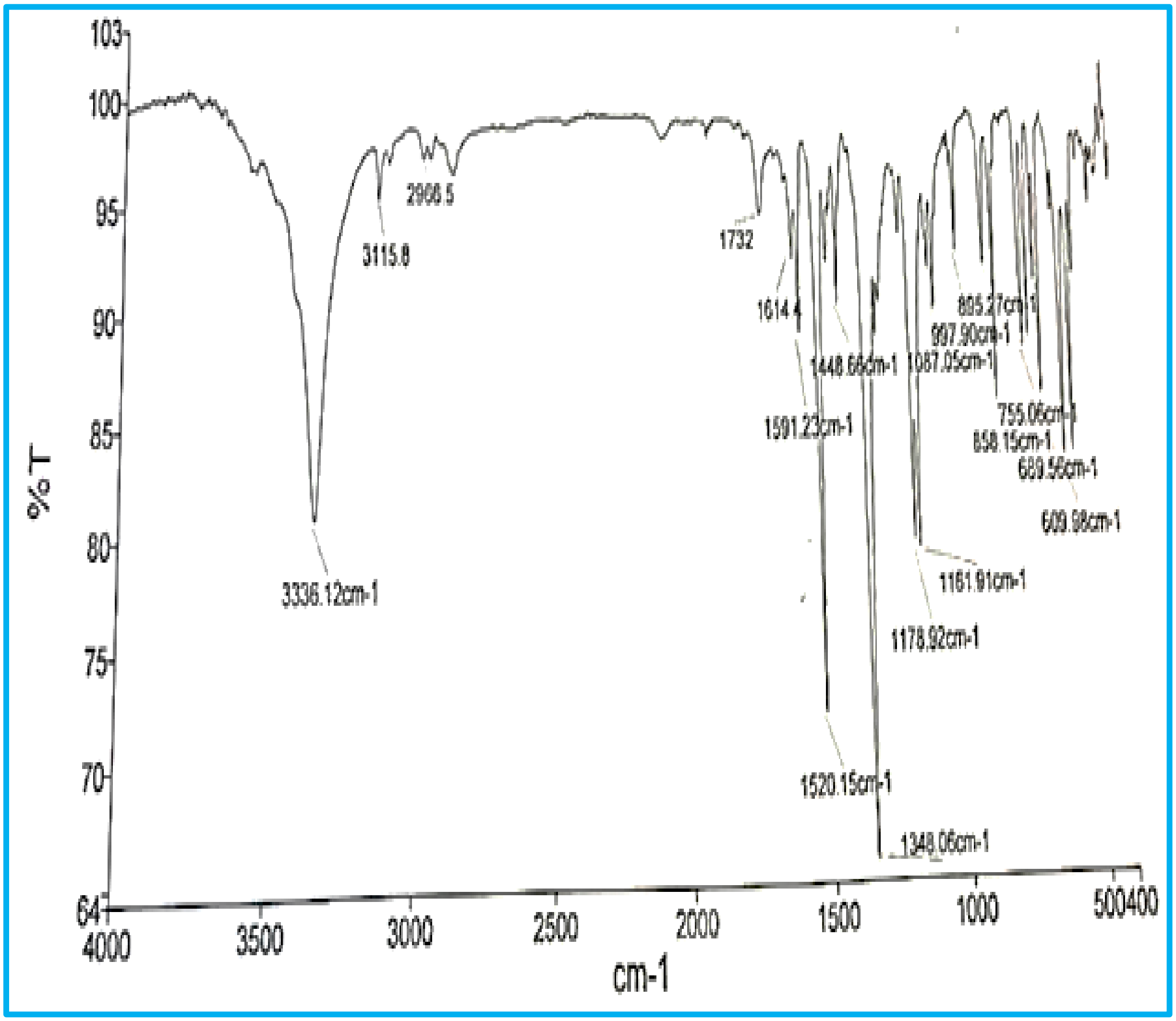

^{1}H NMR spectrum of SA1

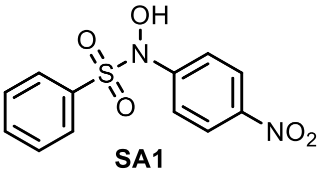

SA1

N-hydroxy-*N*-(4-nitrophenyl)benzenesulfonamide

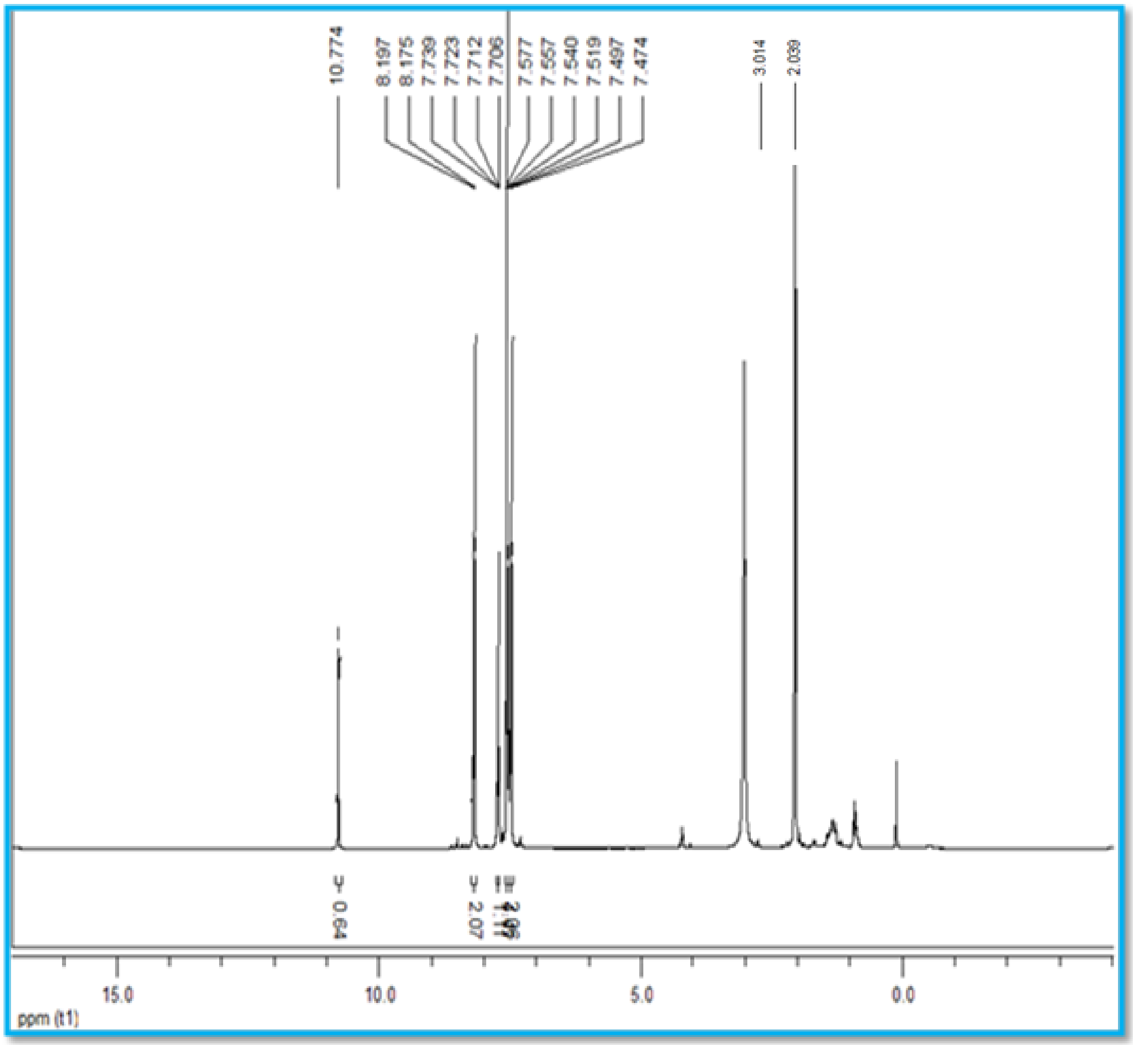

Expanded ^{1}H NMR spectrum of SA1

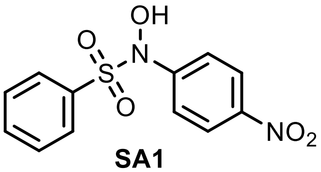

SA1

N-hydroxy-*N*-(4-nitrophenyl)benzenesulfonamide

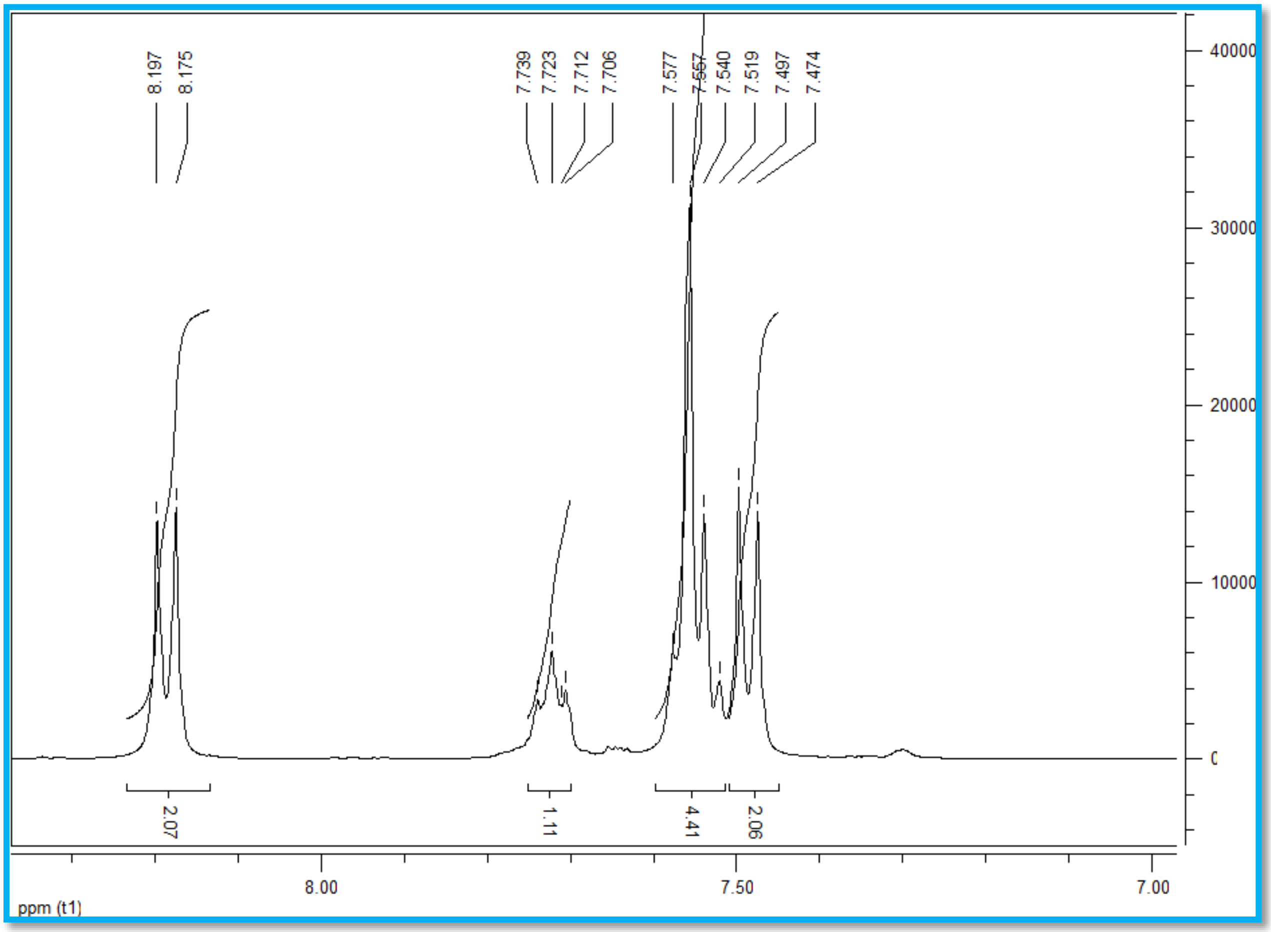

^{13}C NMR spectrum of SA1

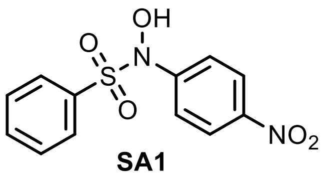

SA1

N-hydroxy-*N*-(4-nitrophenyl)benzenesulfonamide

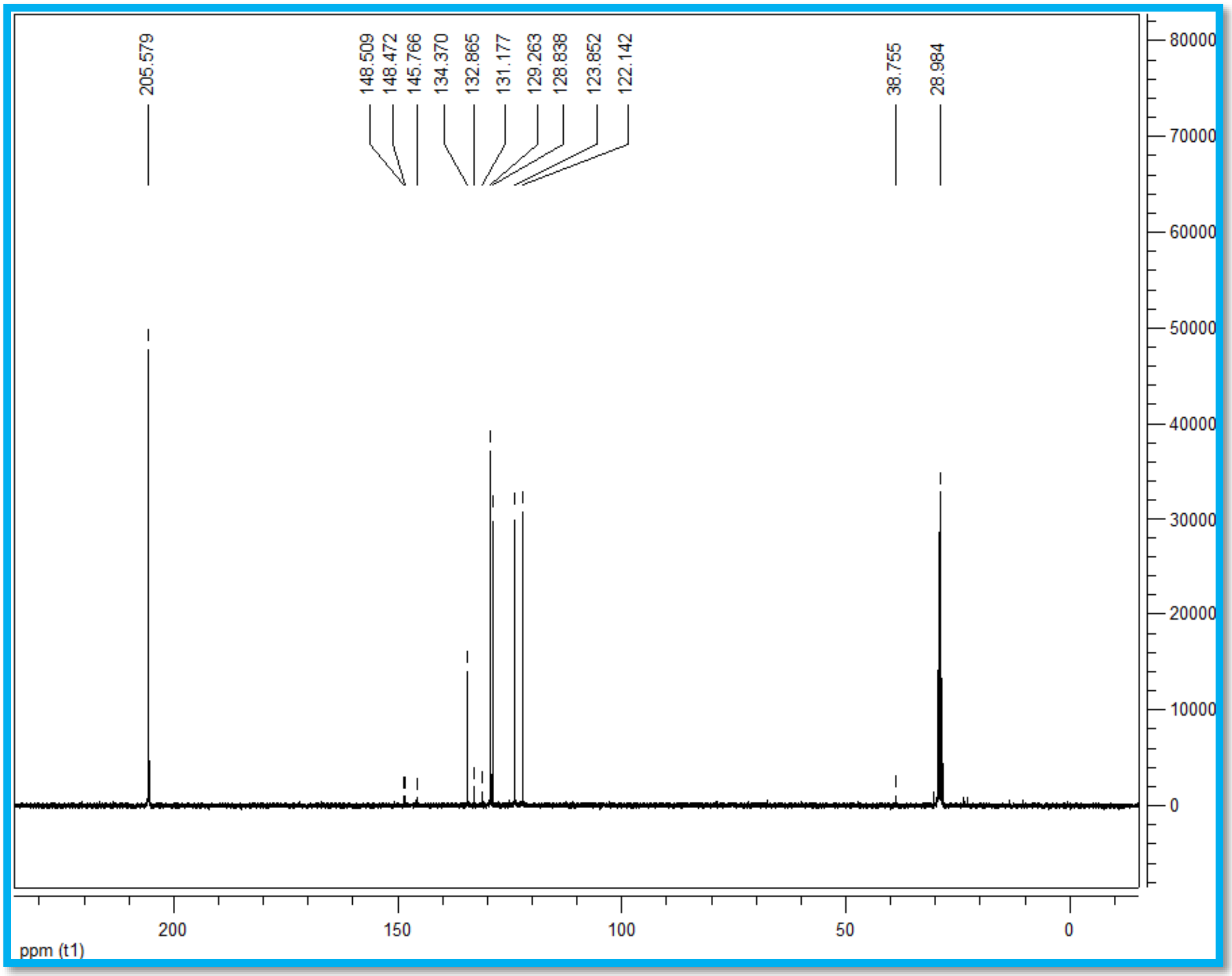

MS spectrum of SA1

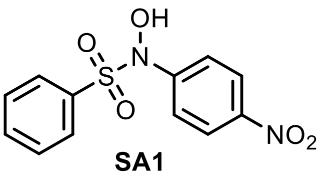

SA1

N-hydroxy-*N*-(4-nitrophenyl)benzenesulfonamide

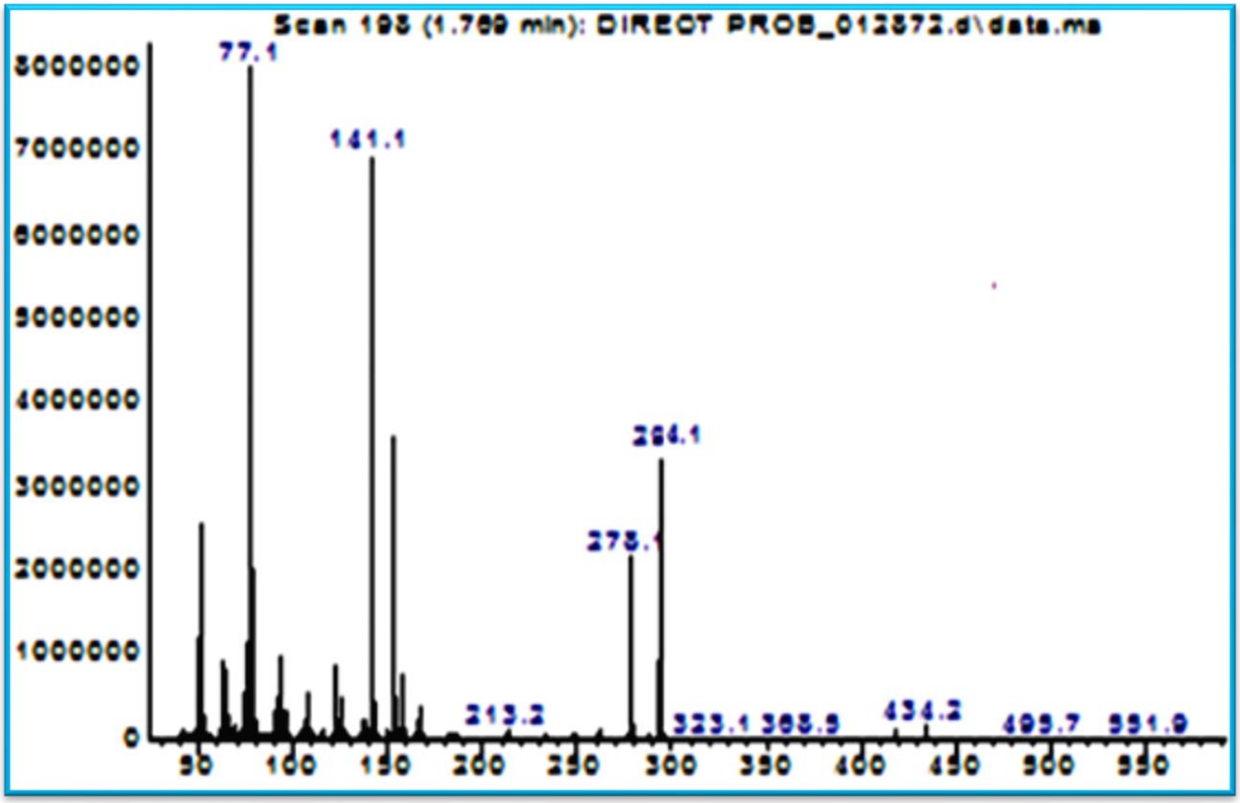

IR spectrum of SA2

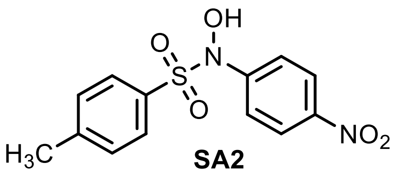

SA2

N-hydroxy-4-methyl-*N*-(4-nitrophenyl)benzenesulfonamide

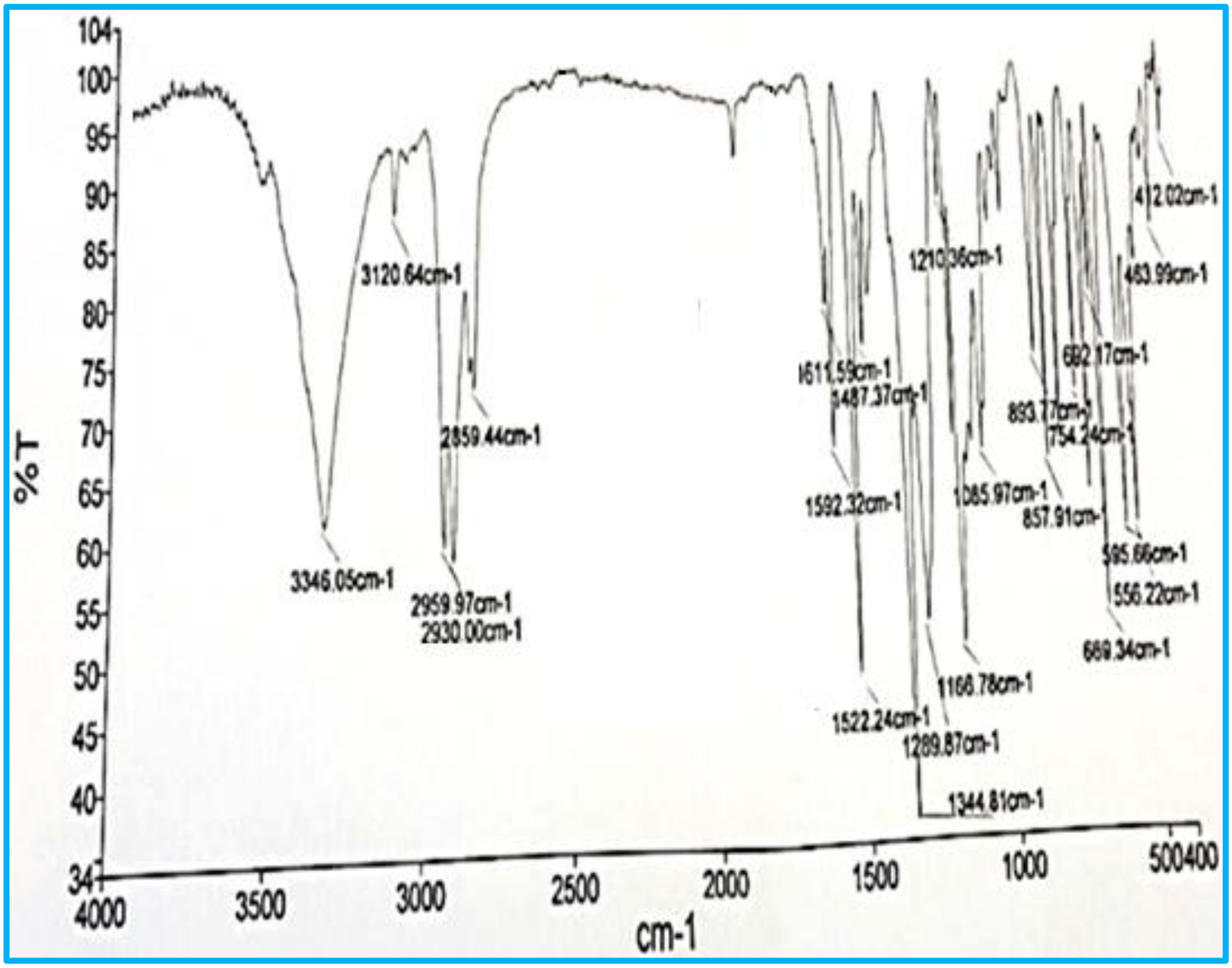

^{1}H NMR spectrum of SA2

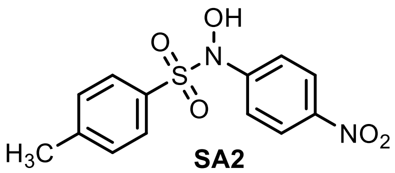

SA2

N-hydroxy-4-methyl-*N*-(4-nitrophenyl)benzenesulfonamide

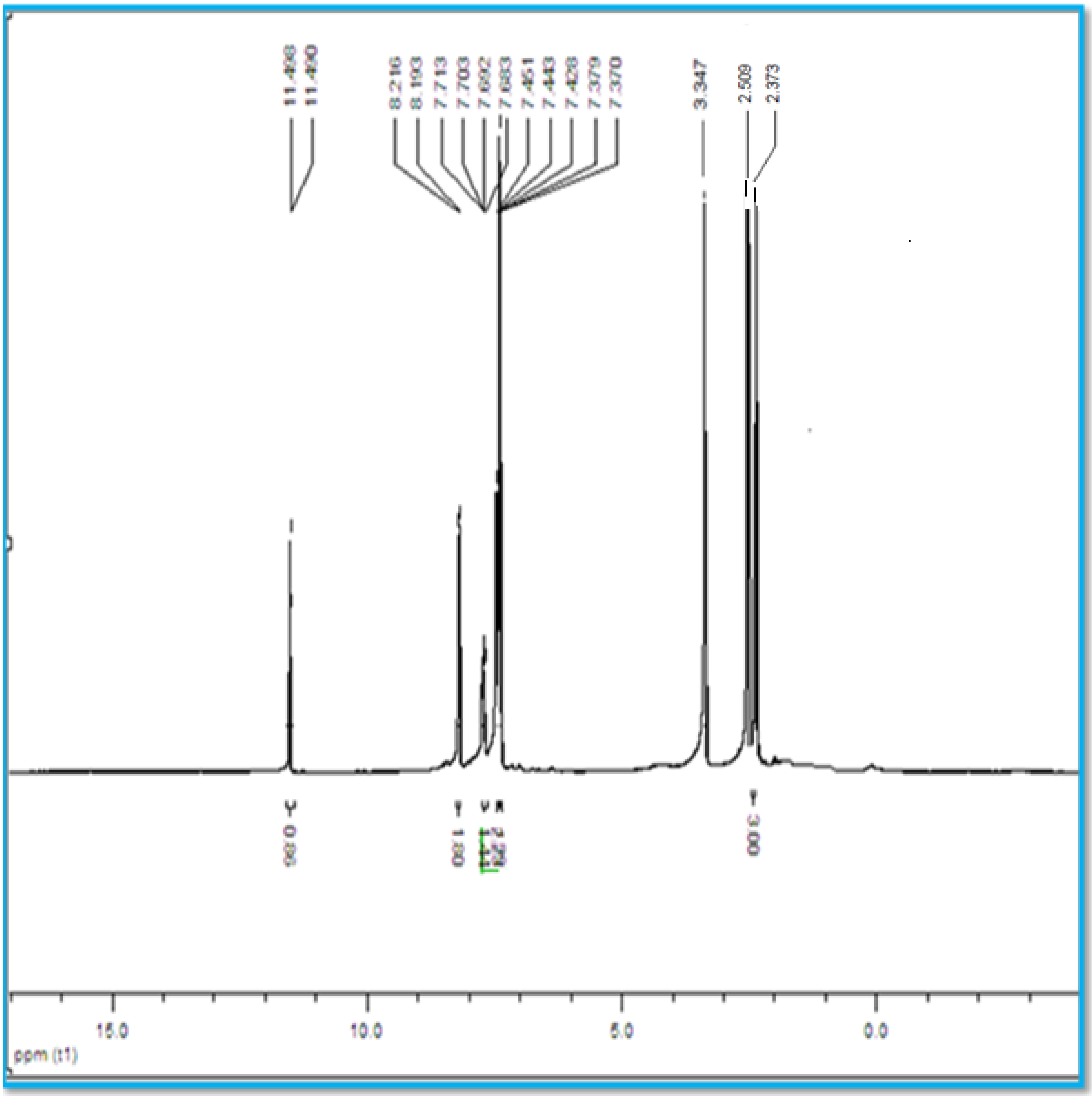

Expanded ^{1}H NMR spectrum of SA2

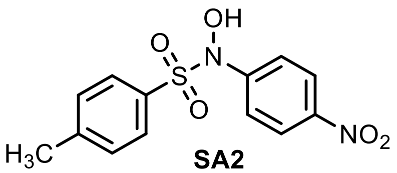

SA2

N-hydroxy-4-methyl-*N*-(4-nitrophenyl)benzenesulfonamide

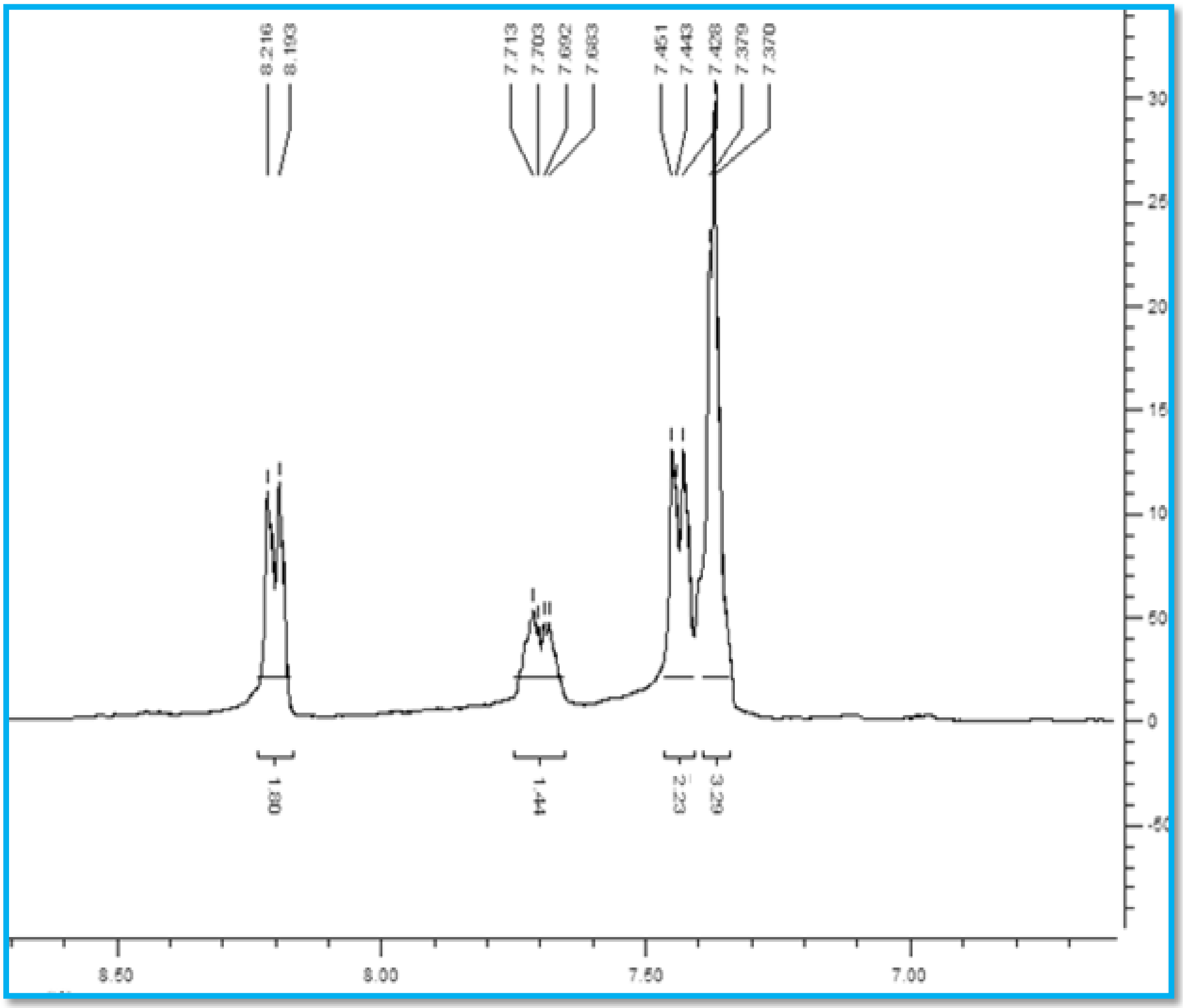

^{13}C NMR spectrum of SA2

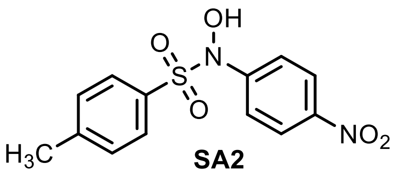

SA2

N-hydroxy-4-methyl-*N*-(4-nitrophenyl)benzenesulfonamide

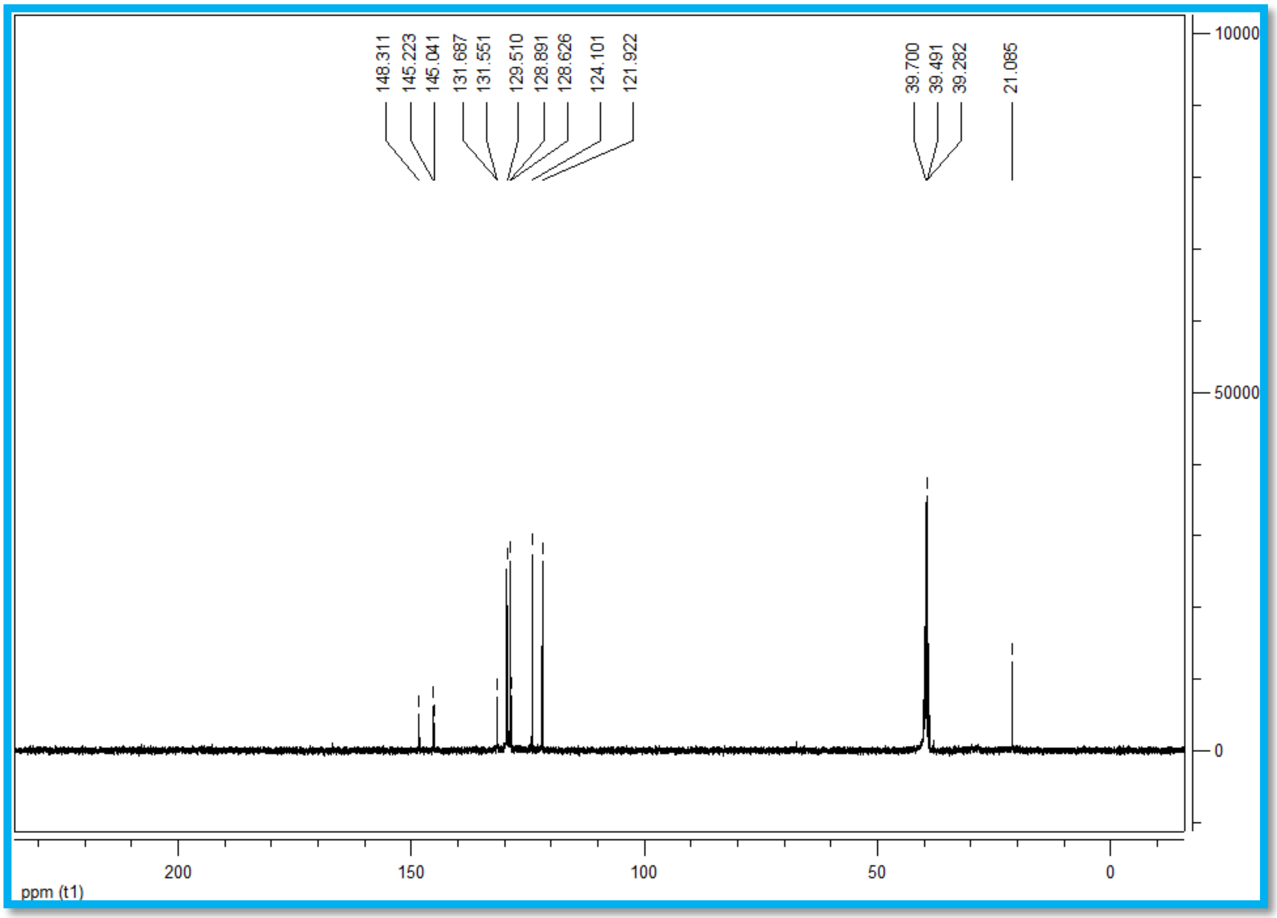

MS spectrum of SA2

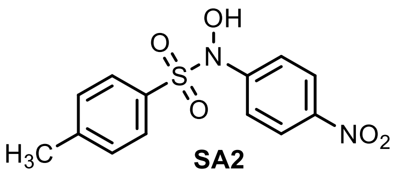

SA2

N-hydroxy-4-methyl-*N*-(4-nitrophenyl)benzenesulfonamide

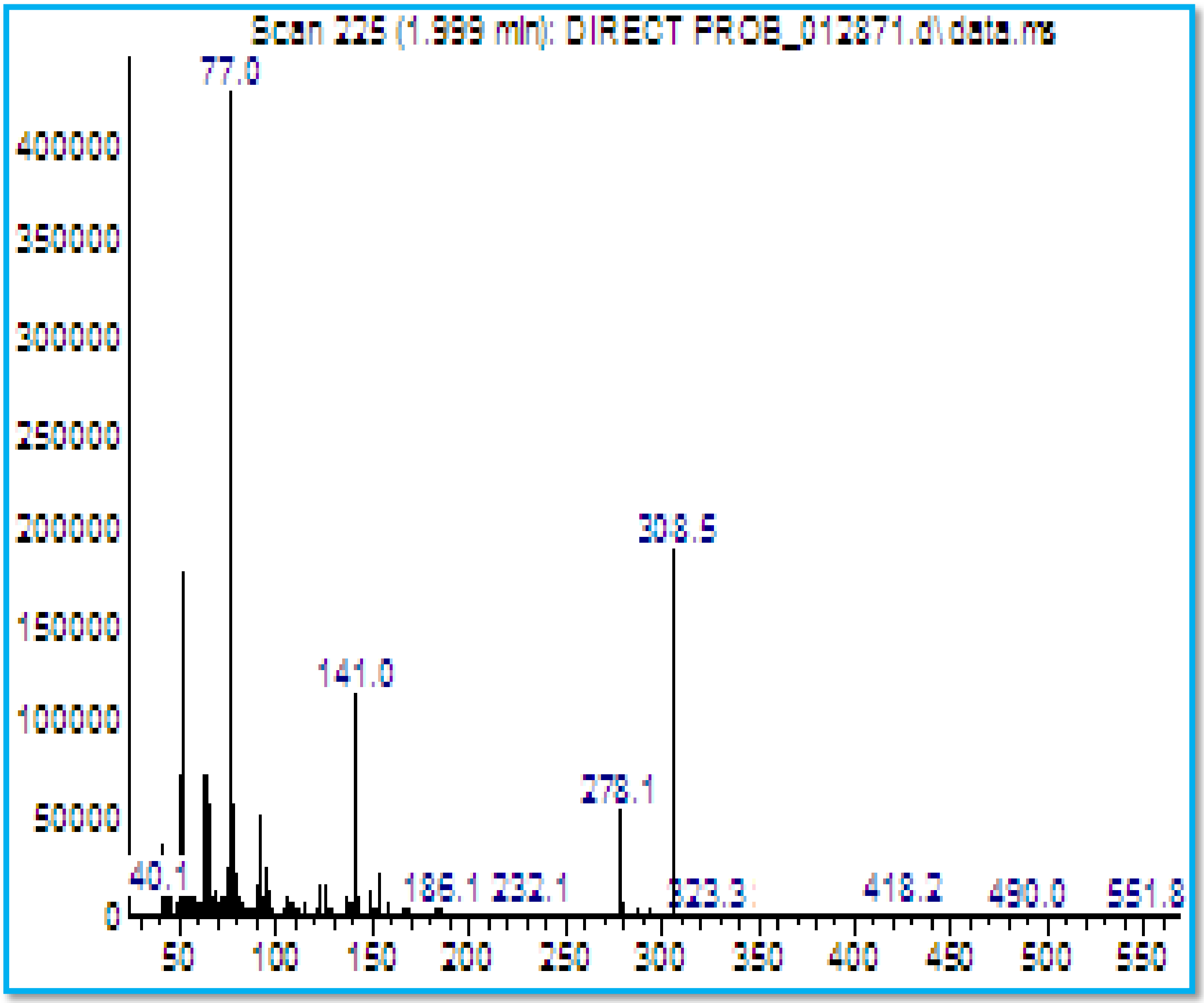

IR spectrum of SA3

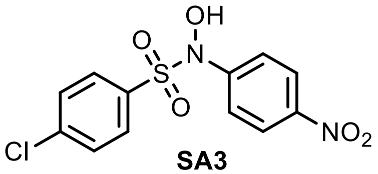

SA3

4-chloro-*N*-hydroxy-*N*-(4-nitrophenyl)benzenesulfonamide

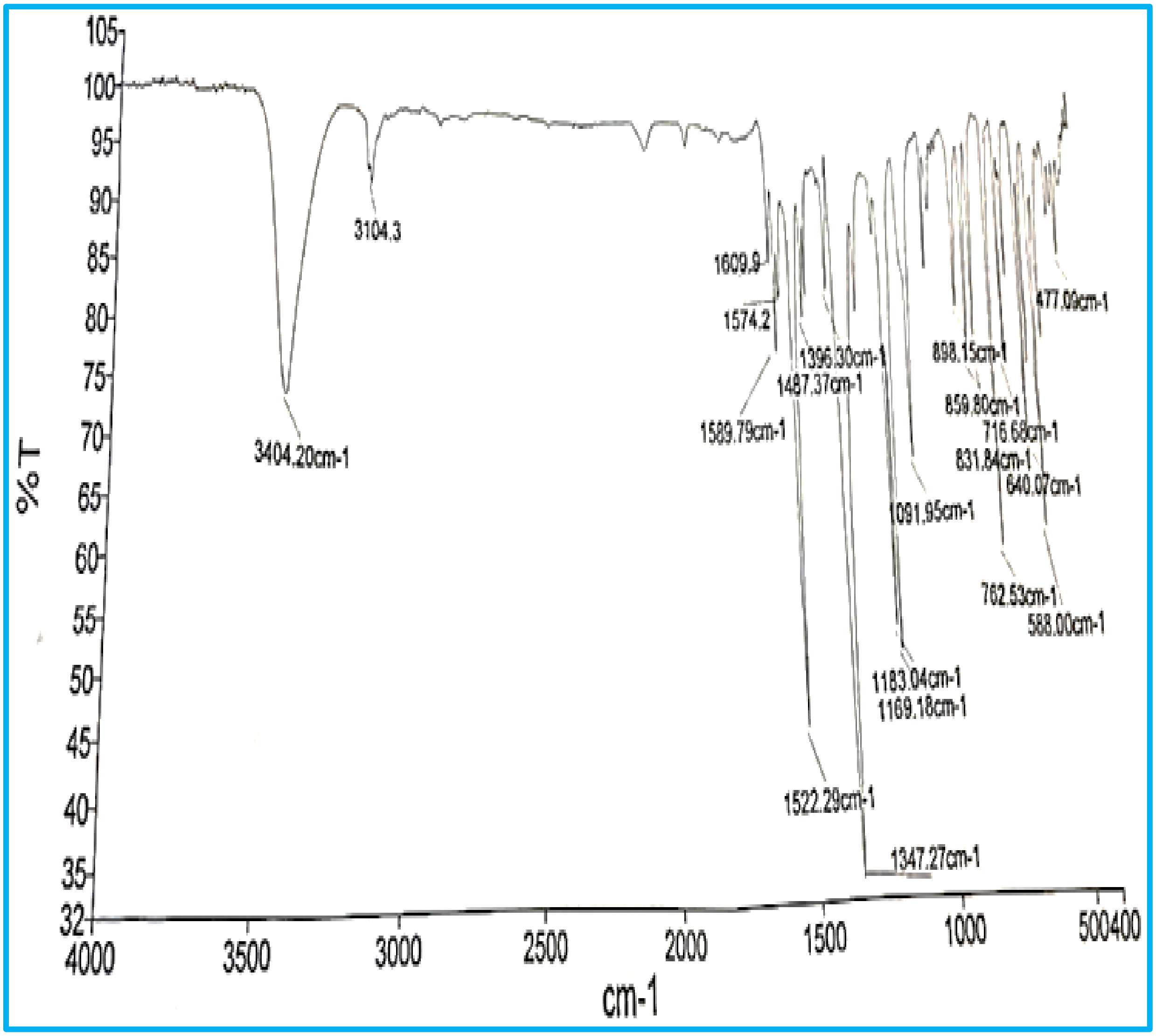

^{1}H NMR spectrum of SA3

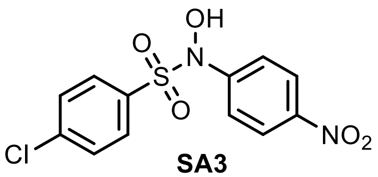

SA3

4-chloro-*N*-hydroxy-*N*-(4-nitrophenyl)benzenesulfonamide

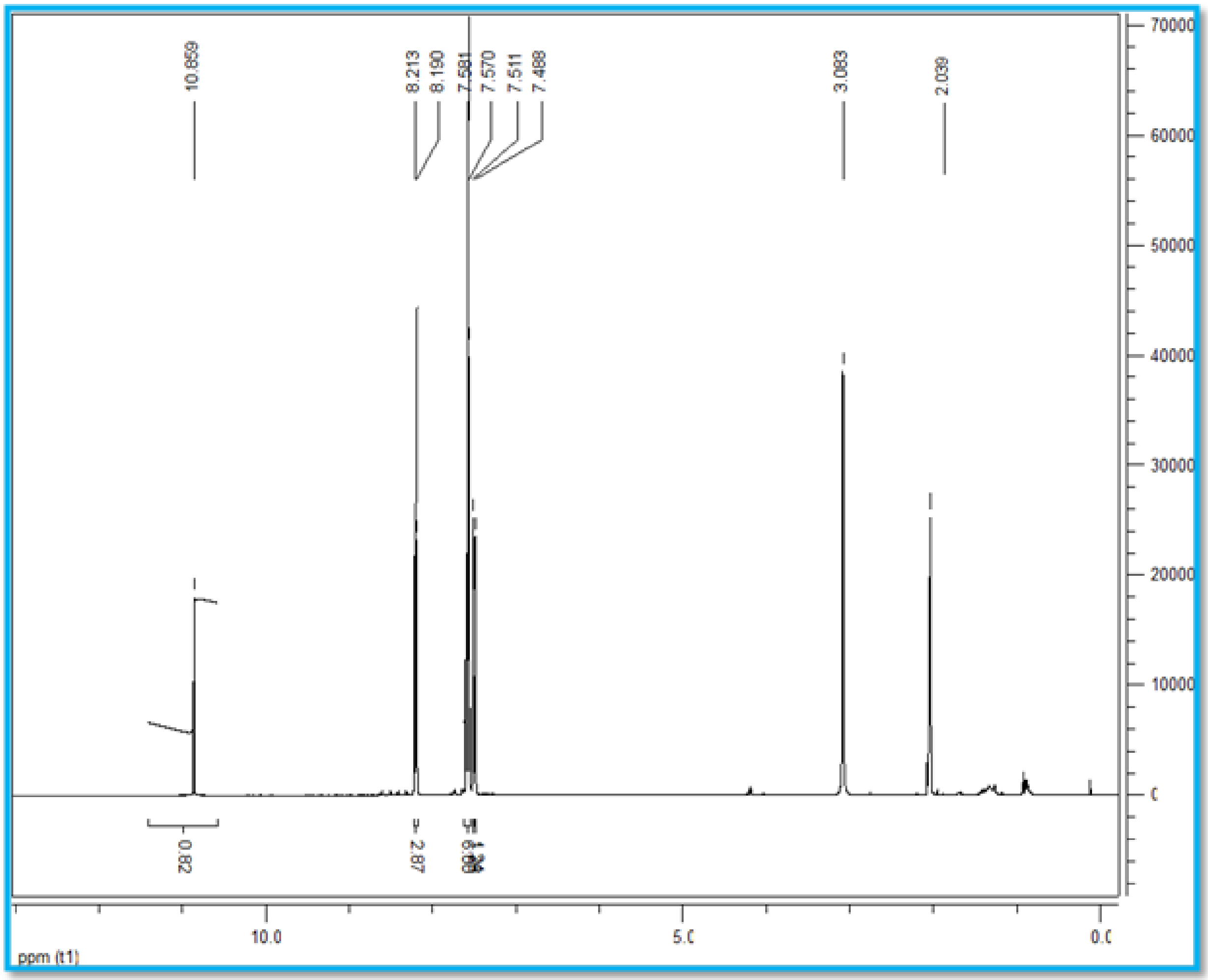

Expanded ^{1}H NMR spectrum of SA3

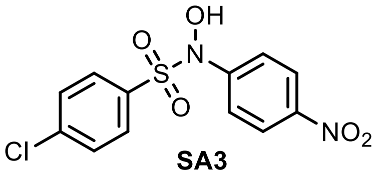

SA3

4-chloro-*N*-hydroxy-*N*-(4-nitrophenyl)benzenesulfonamide

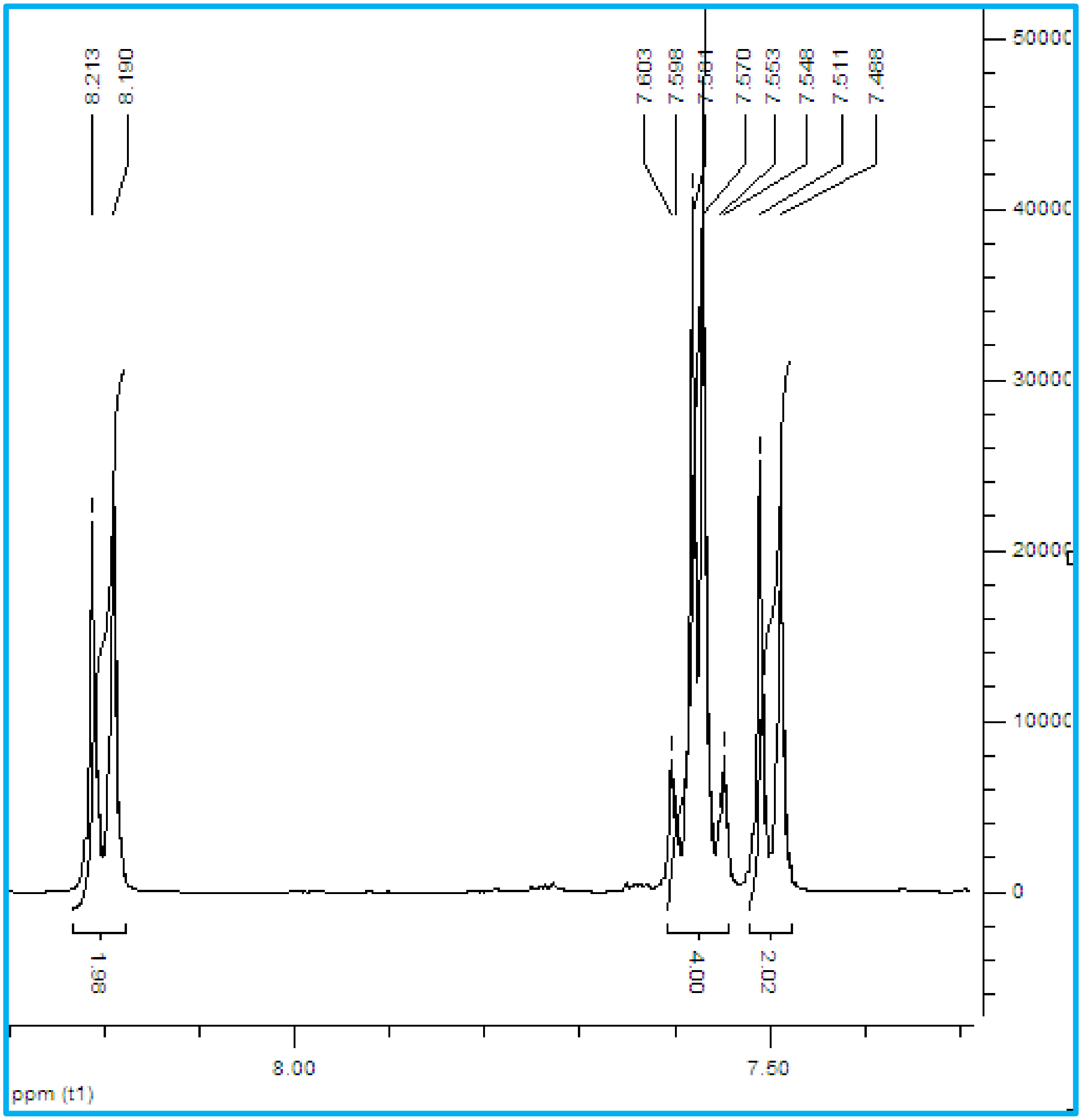

^{13}C NMR spectrum of SA3

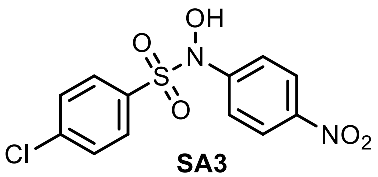

SA3

4-chloro-*N*-hydroxy-*N*-(4-nitrophenyl)benzenesulfonamide

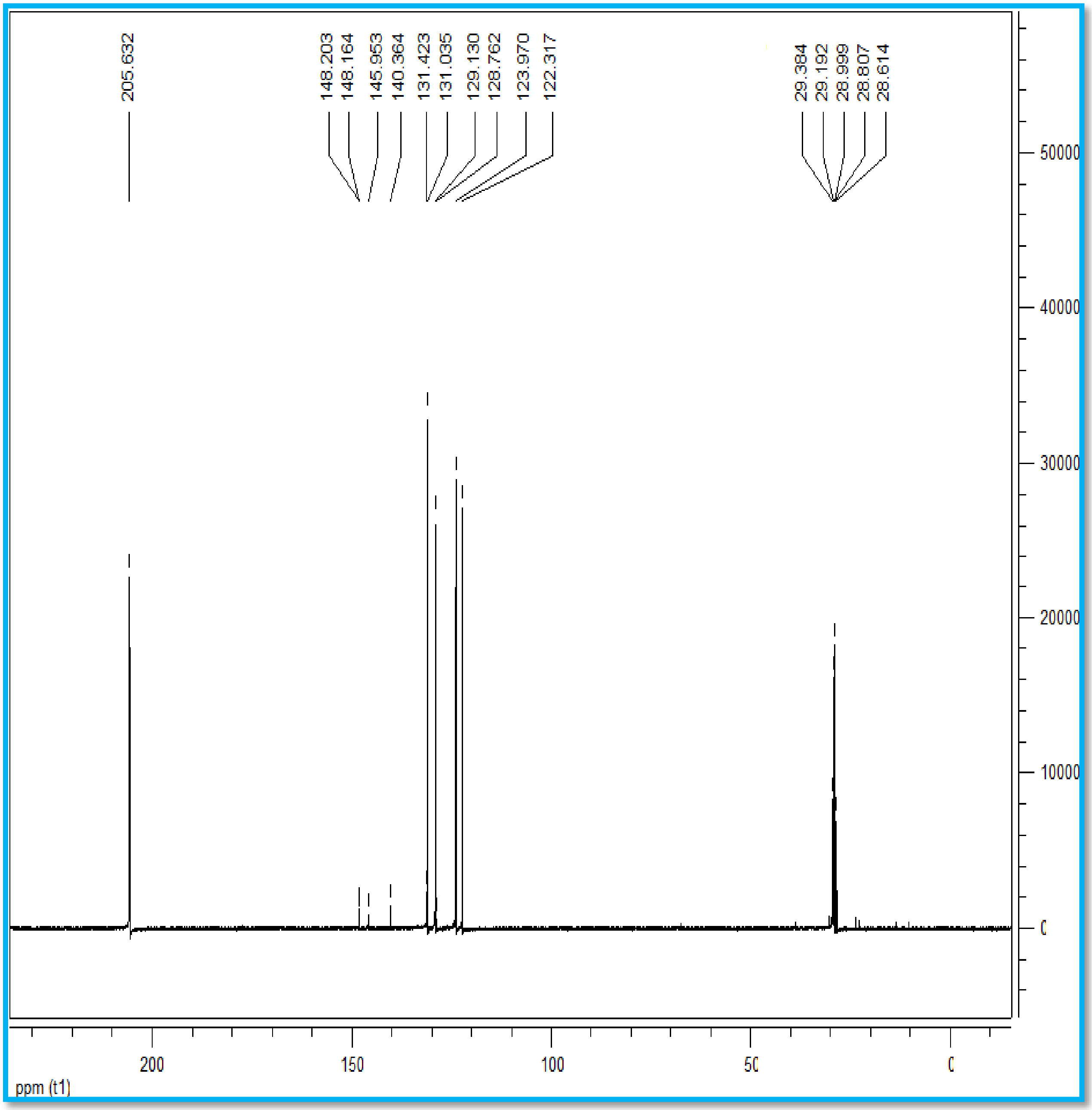

MS spectrum of SA3

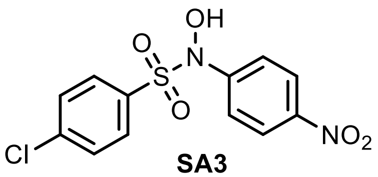

SA3

4-chloro-*N*-hydroxy-*N*-(4-nitrophenyl)benzenesulfonamide

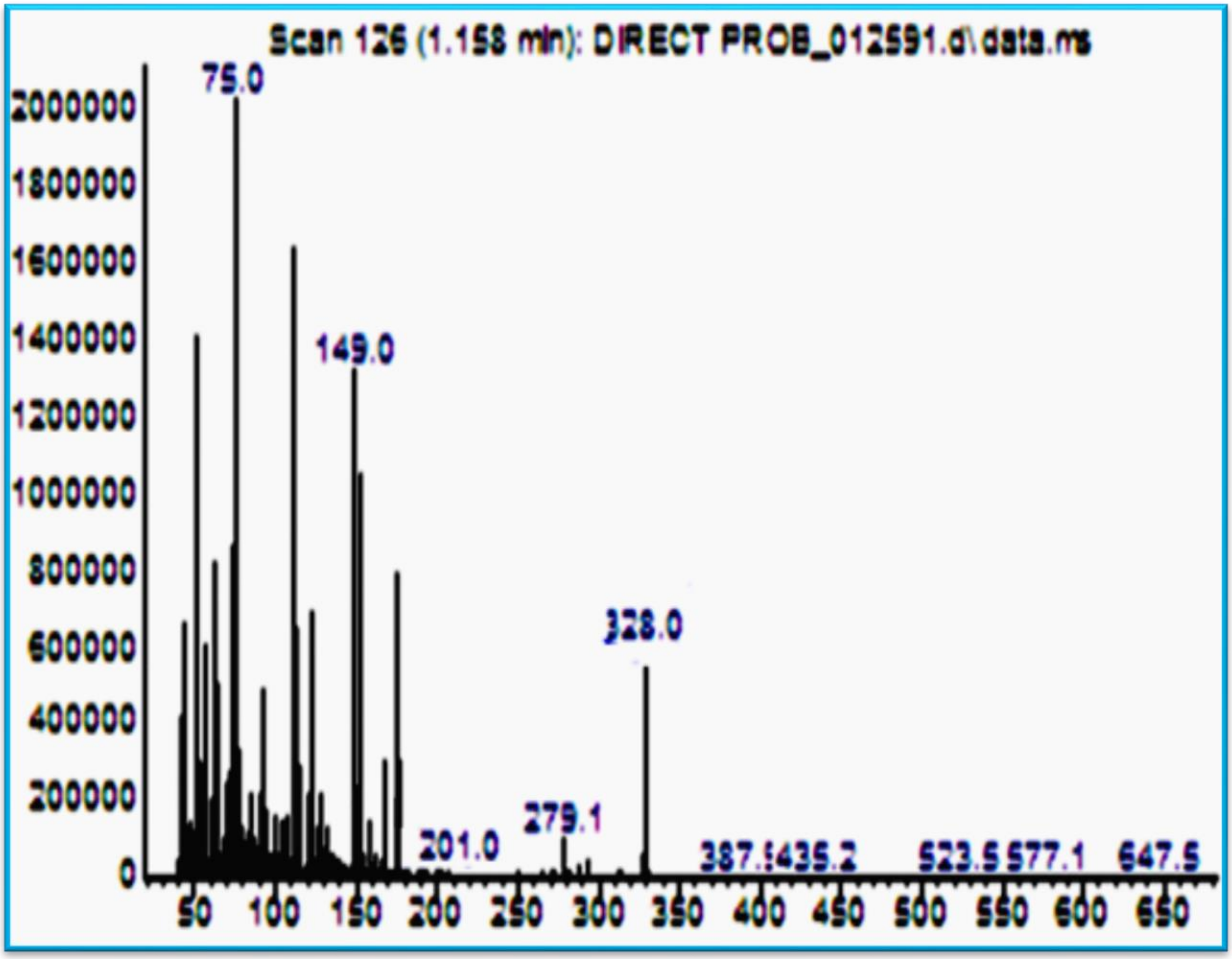

IR spectrum of NS1

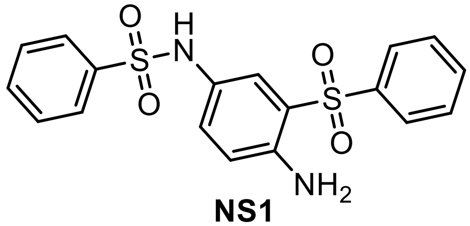

NS1

N-(4-amino-3-(phenylsulfonyl)phenyl)benzenesulfonamide

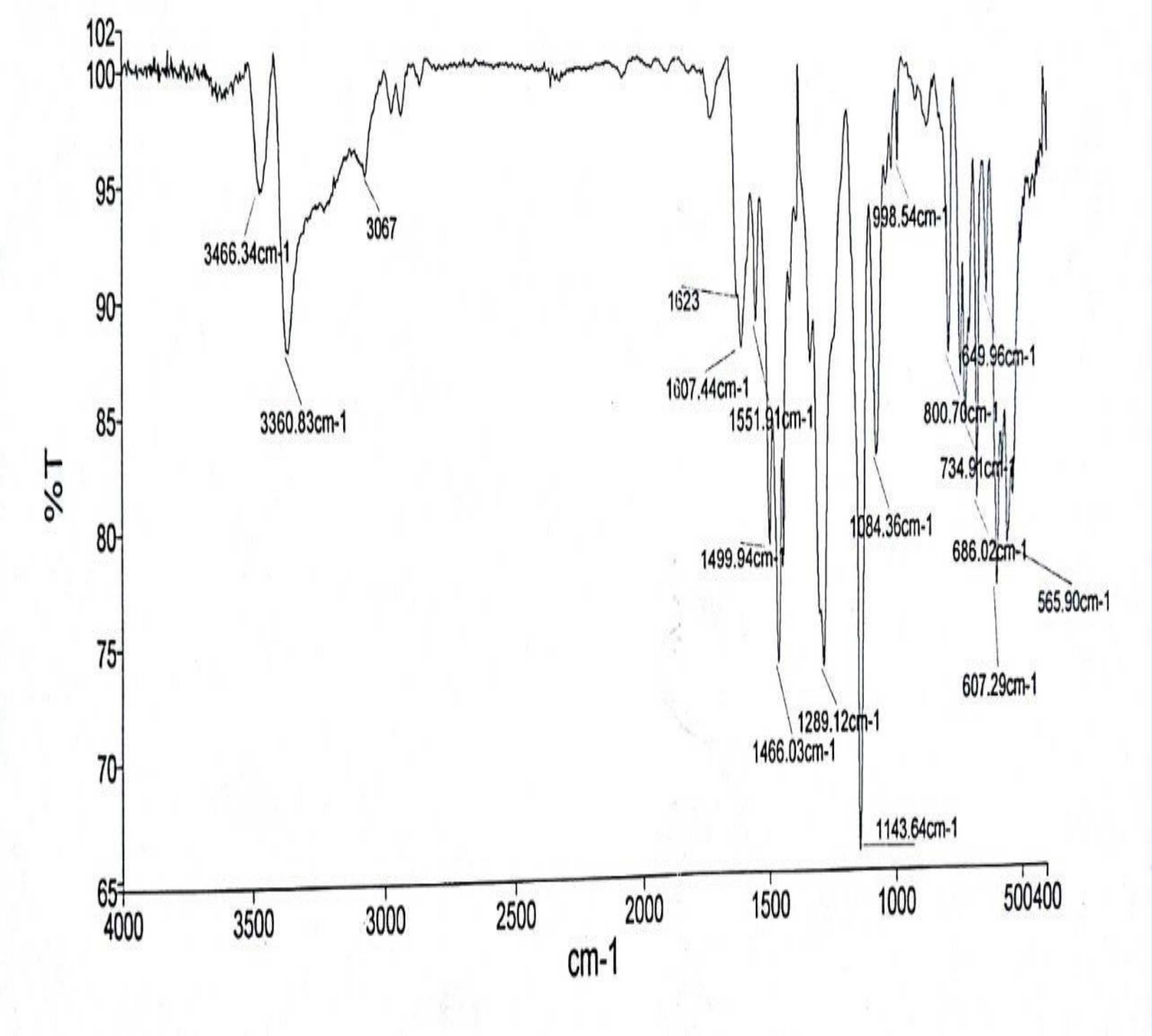

^{1}HNMR spectrum of NS1

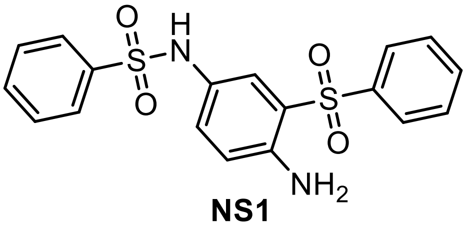

N-(4-amino-3-(phenylsulfonyl)phenyl)benzenesulfonamide

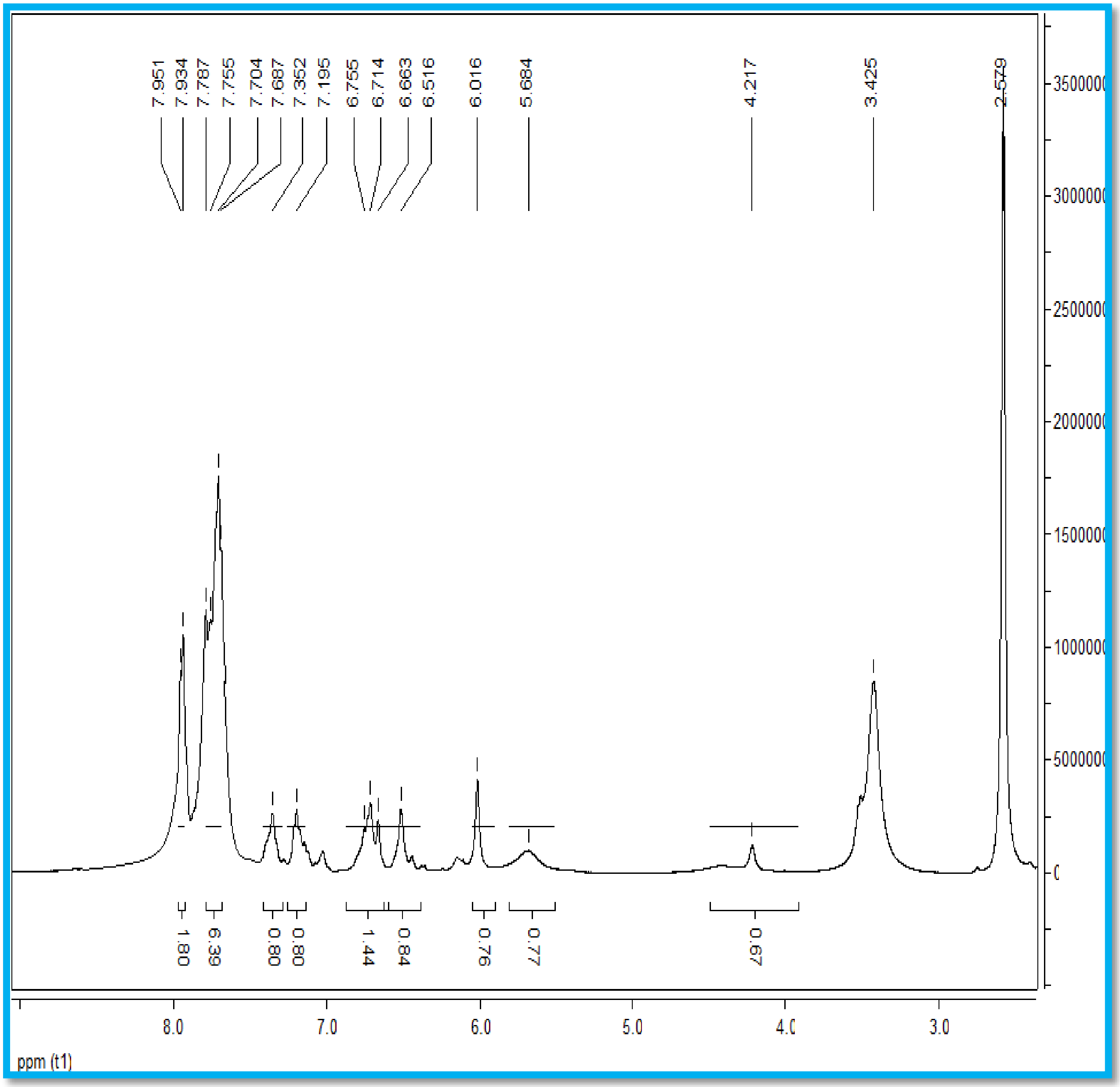

^{13}C NMR spectrum of NS1

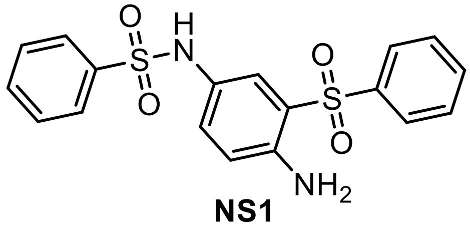

NS1

N-(4-amino-3-(phenylsulfonyl)phenyl)benzenesulfonamide

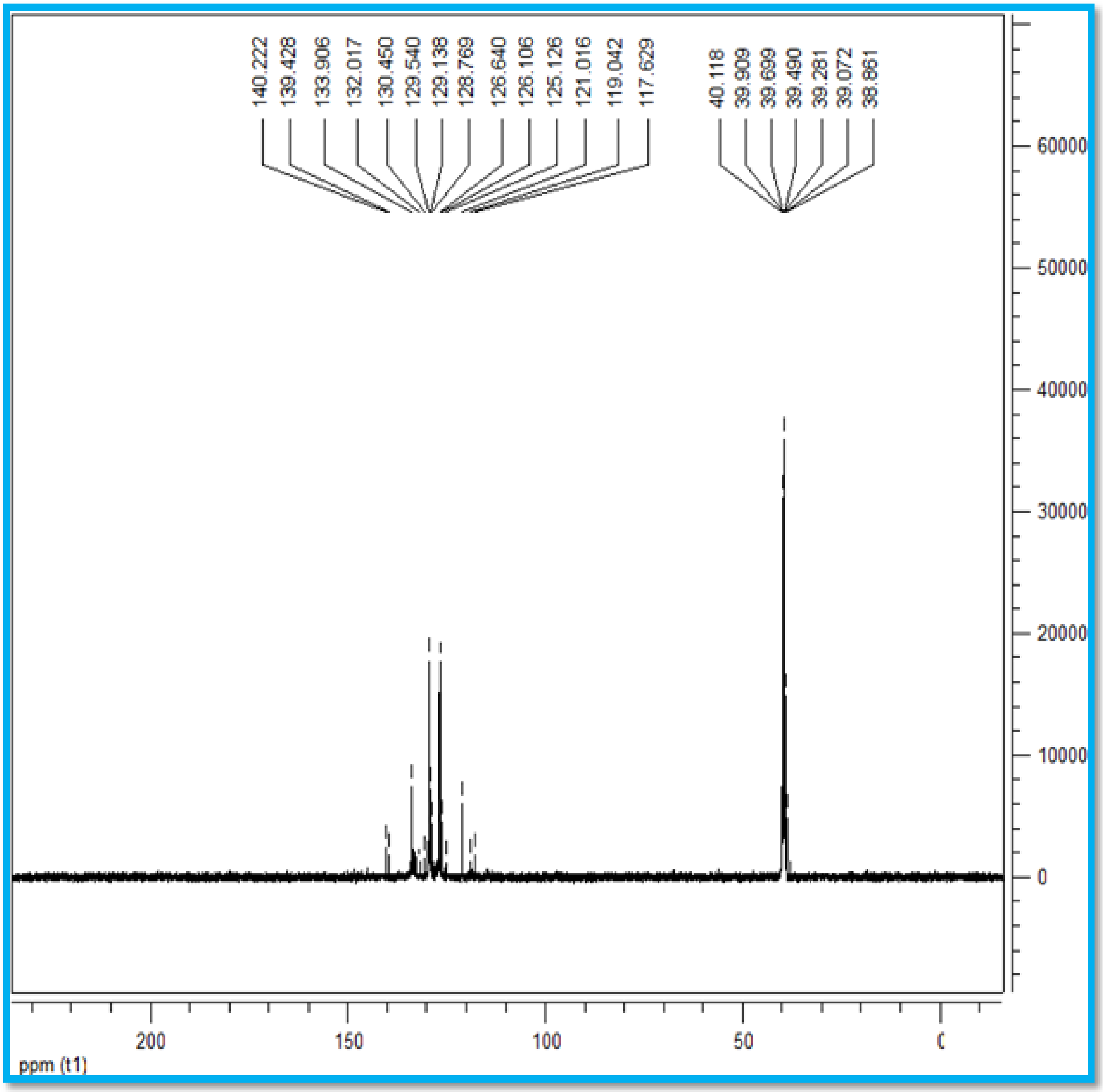

^{13}C NMR Expanded spectrum of NS1

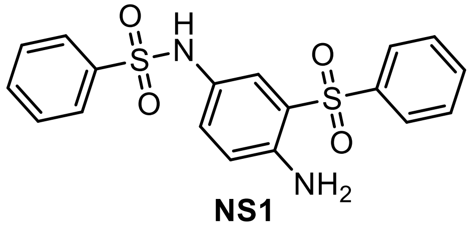

N-(4-amino-3-(phenylsulfonyl)phenyl)benzenesulfonamide

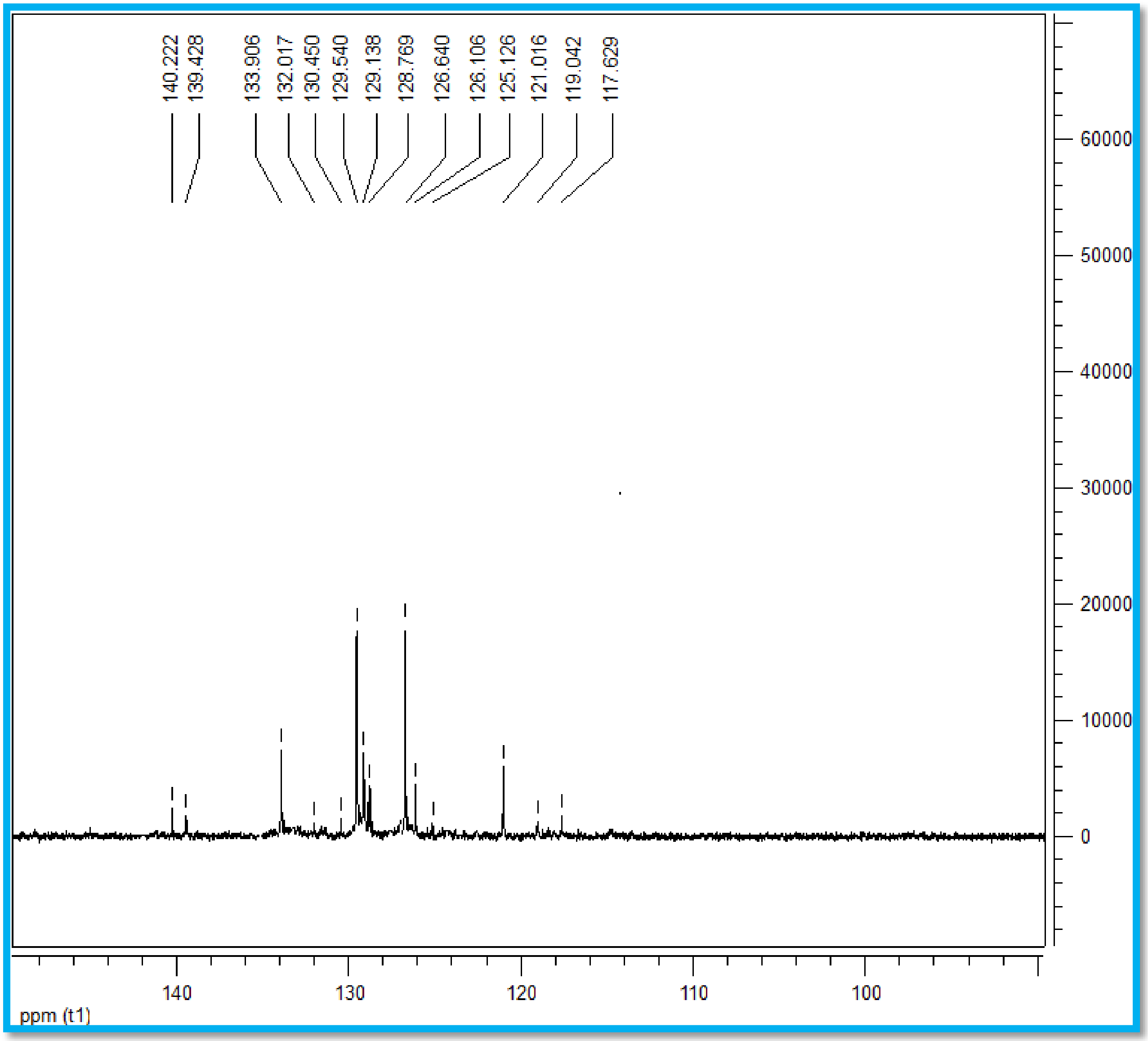

MS spectrum of NS1

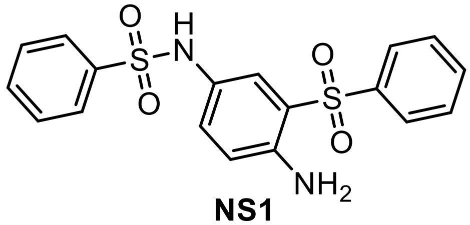

NS1

N-(4-amino-3-(phenylsulfonyl)phenyl)benzenesulfonamide

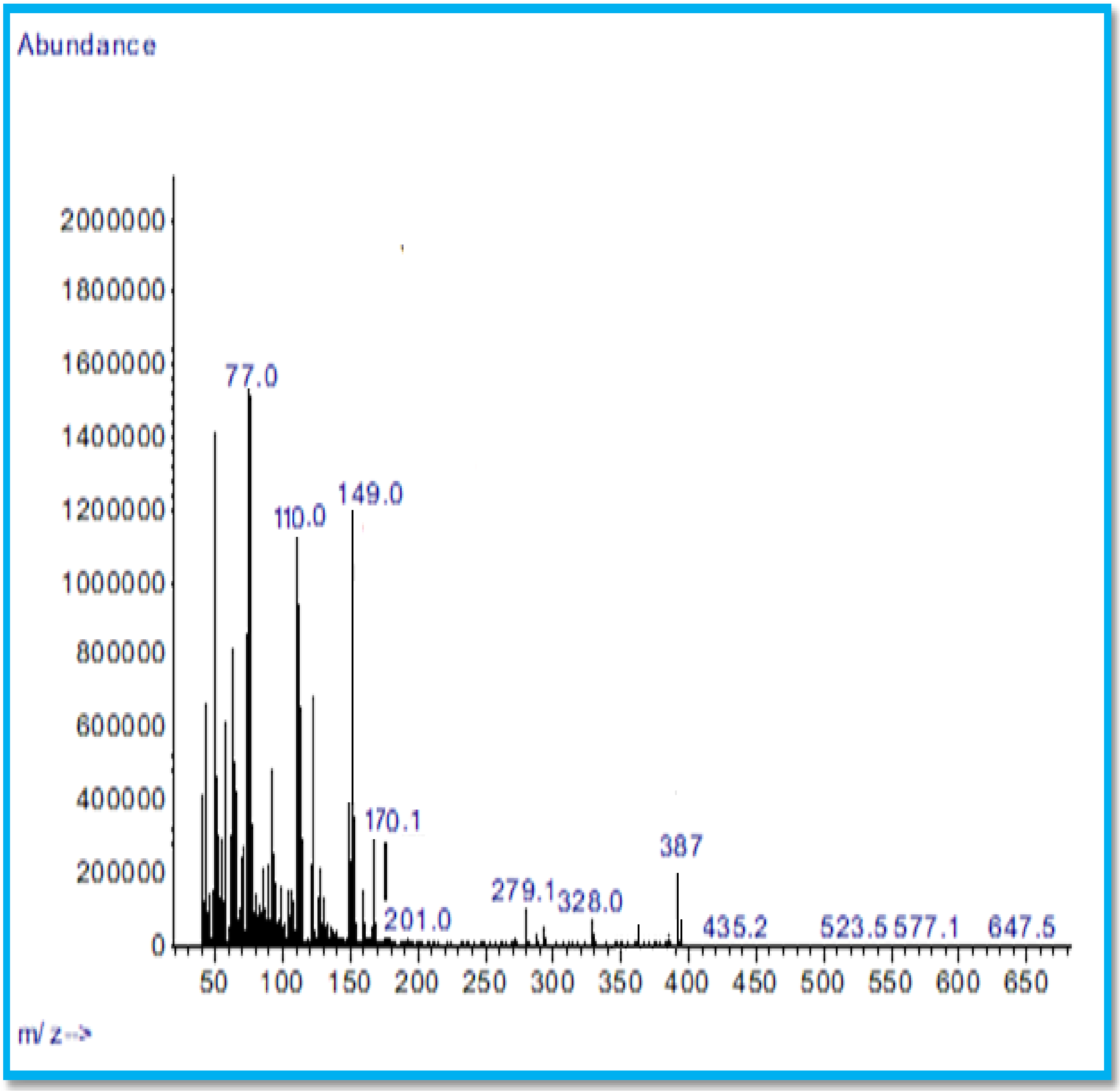

IR spectrum of NS2

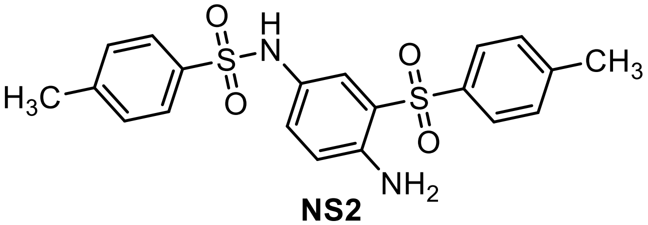

NS2

N-(4-amino-3-tosylphenyl)-4-methylbenzenesulfonamide)

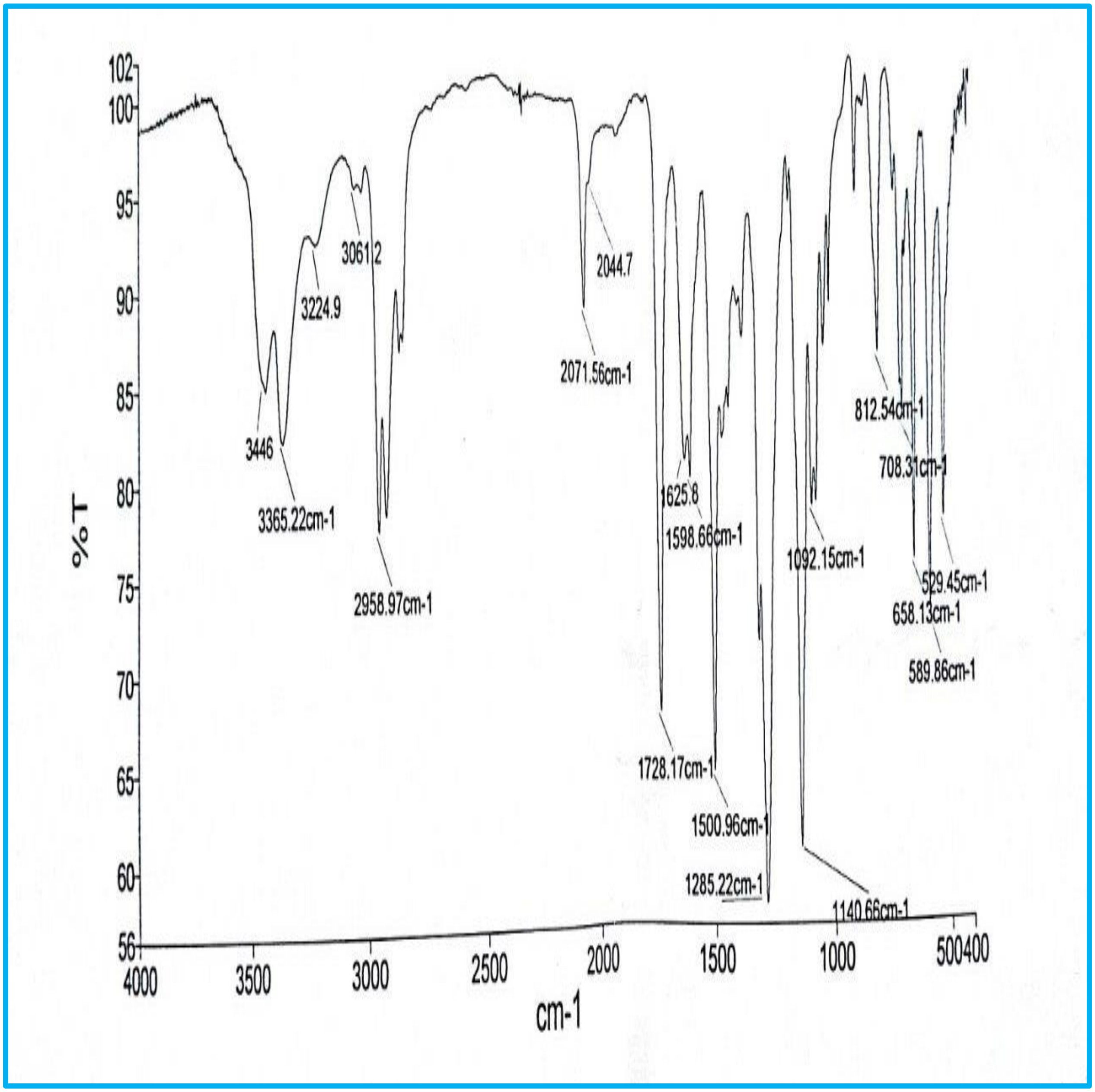

^{1}H NMR spectrum of NS2

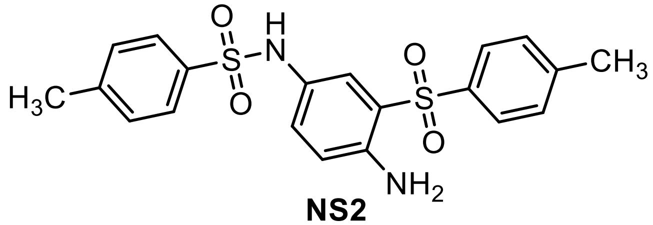

NS2

N-(4-amino-3-tosylphenyl)-4-methylbenzenesulfonamide)

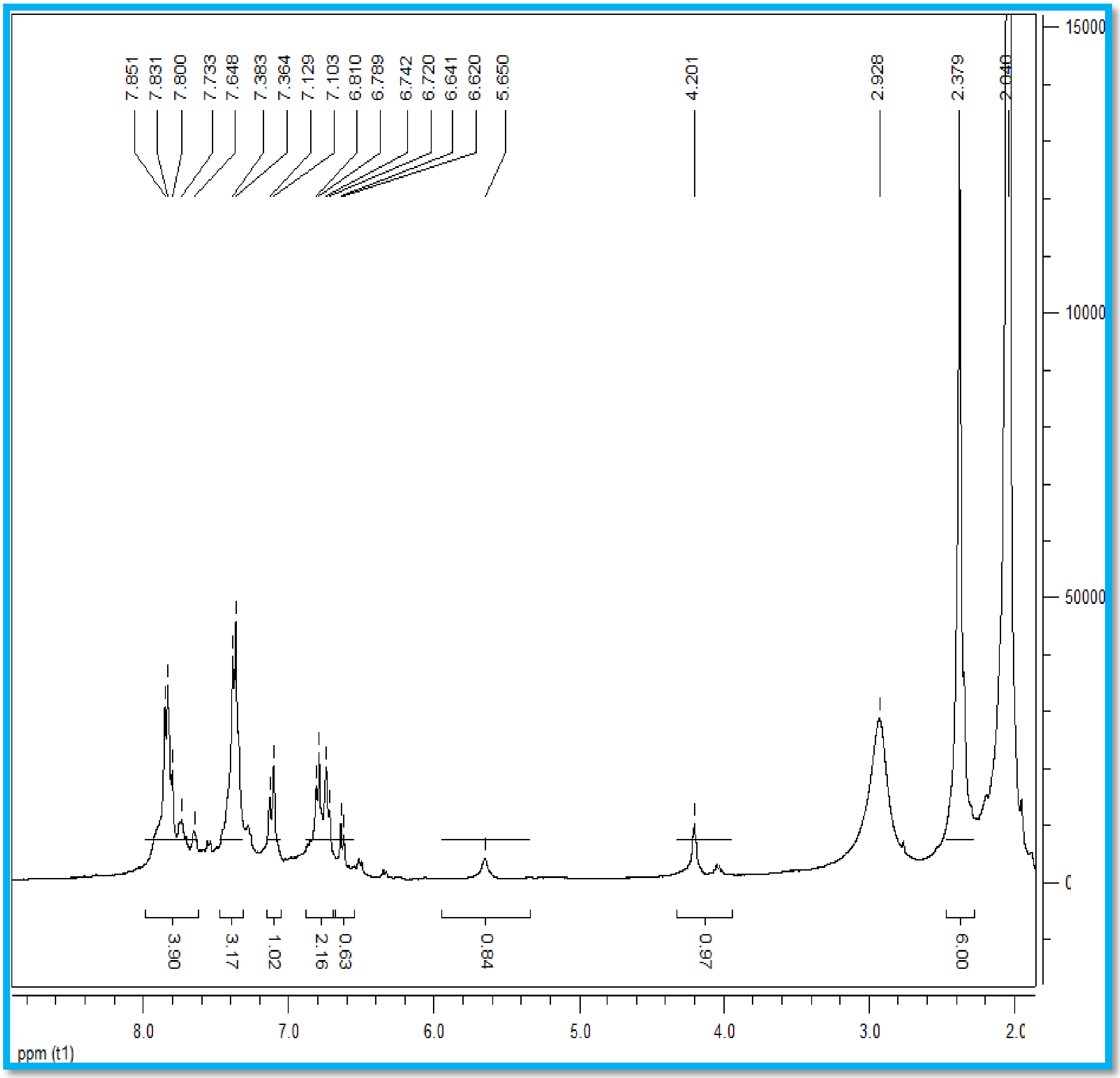

^{13}C NMR spectrum of NS2

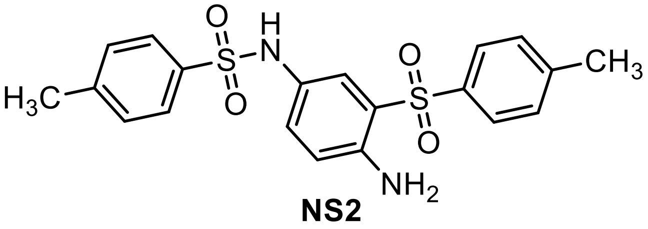

N-(4-amino-3-tosylphenyl)-4-methylbenzenesulfonamide)

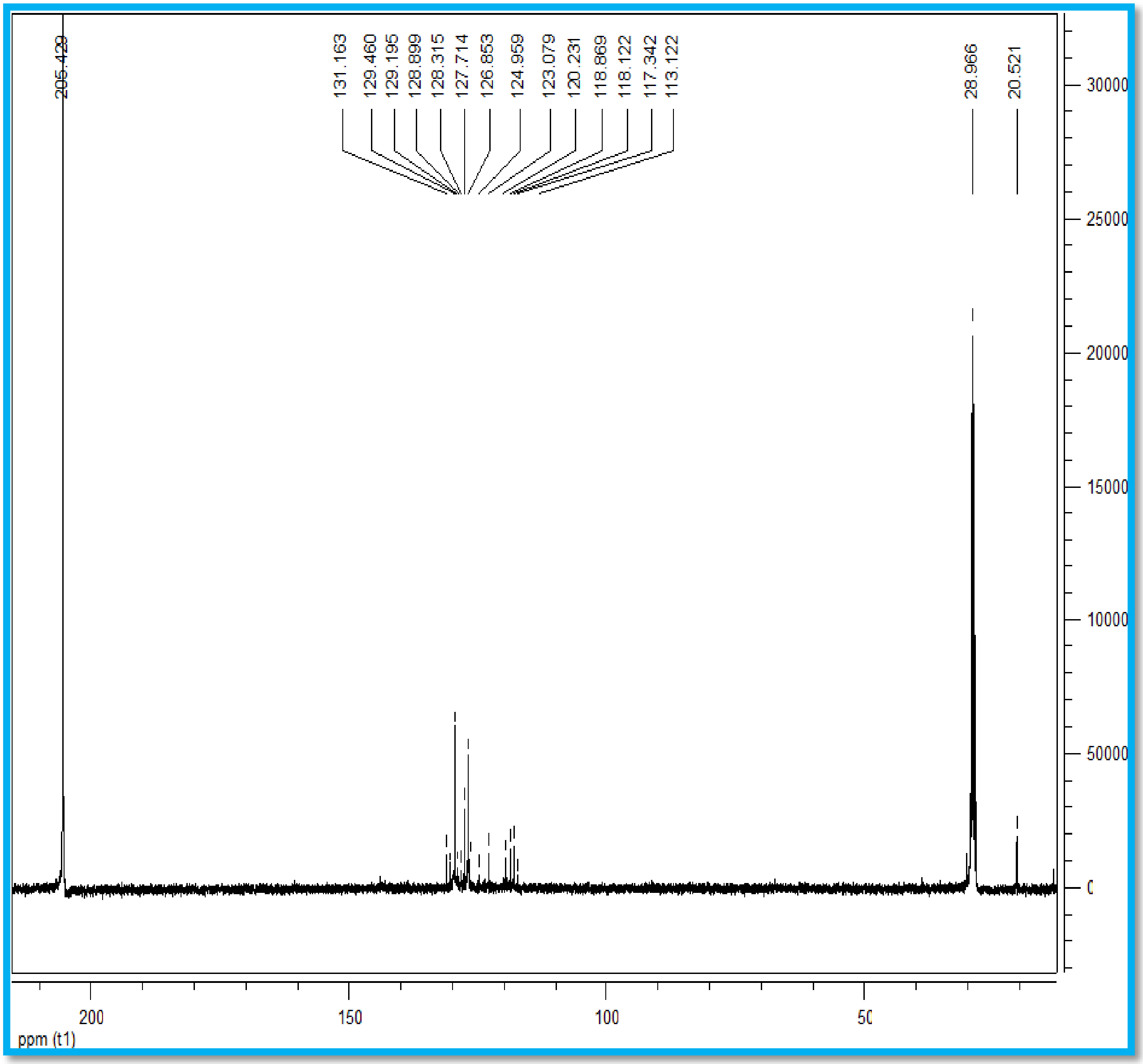

MS spectrum of NS2

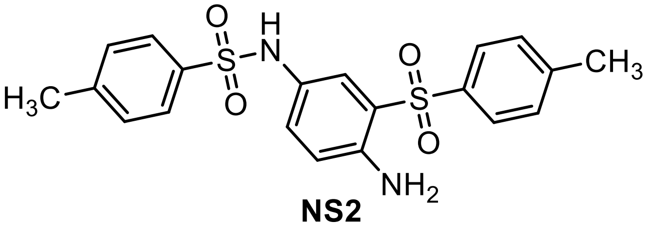

N-(4-amino-3-tosylphenyl)-4-methylbenzenesulfonamide)

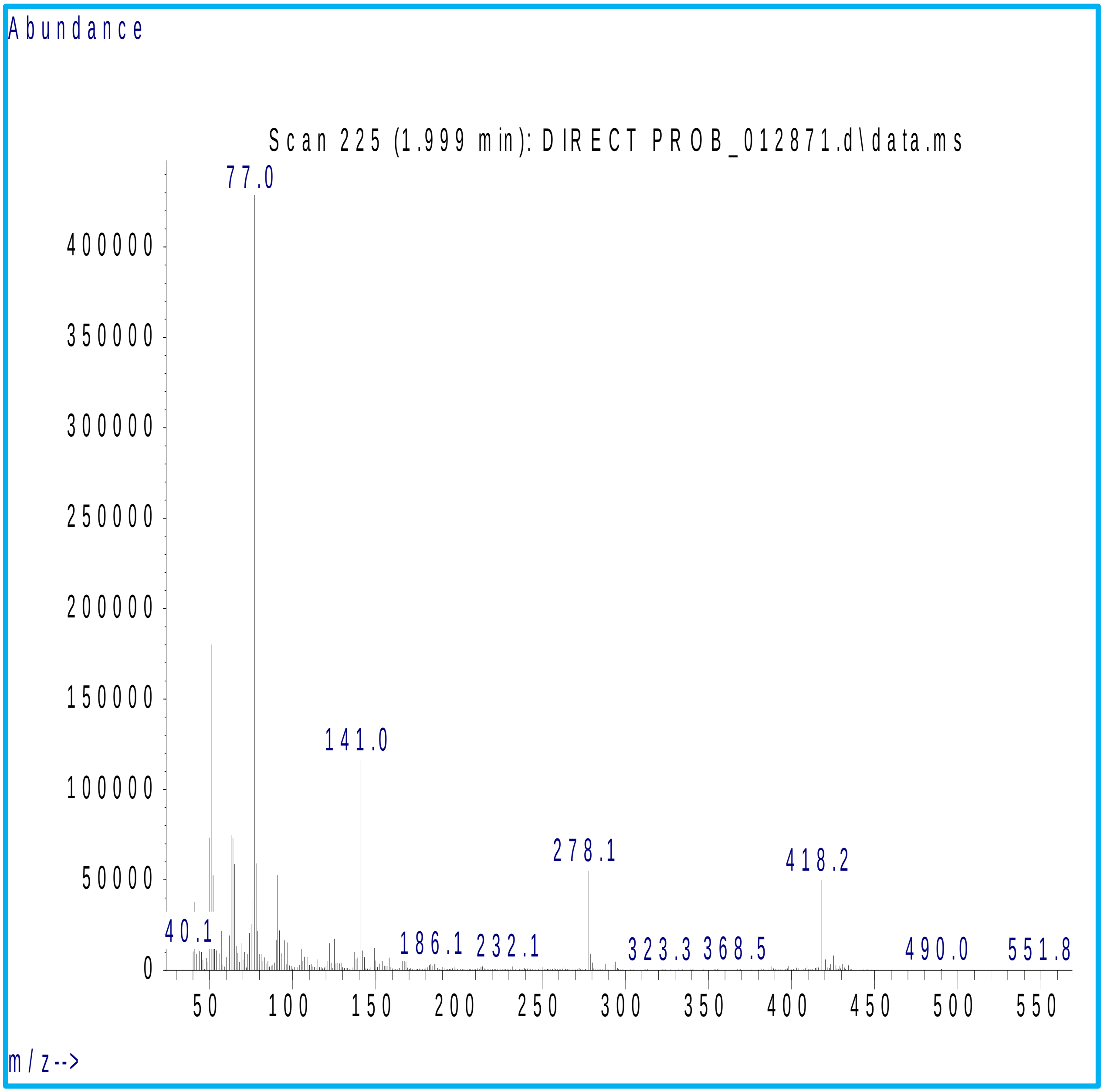

IR spectrum of NS3

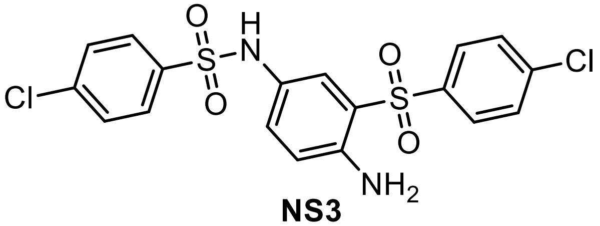

NS3

N-(4-amino-3-(4-chlorophenylsulfonyl)phenyl)-4-chlorobenzenesulfonamide

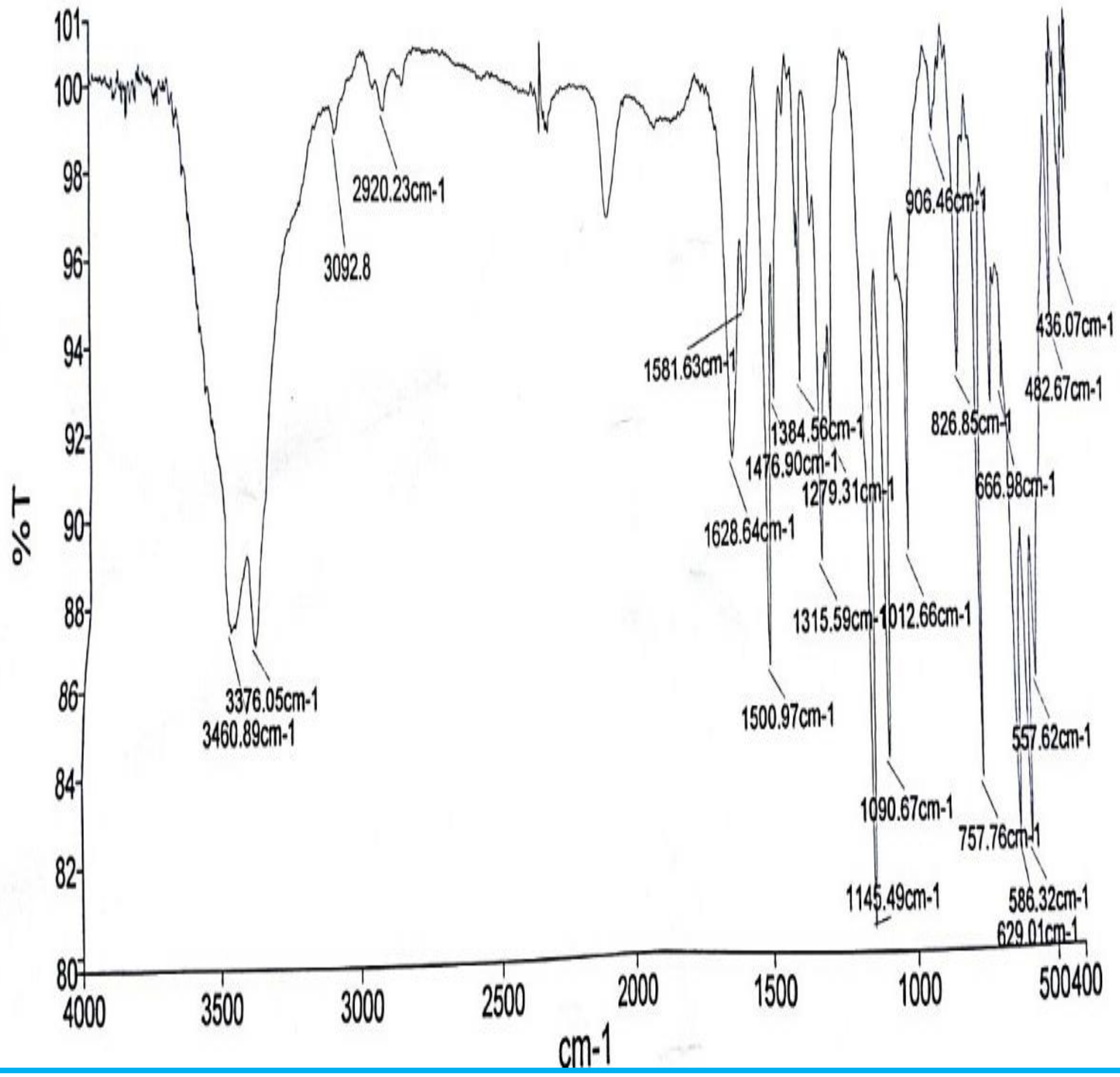

^{1}H NMR spectrum of NS3

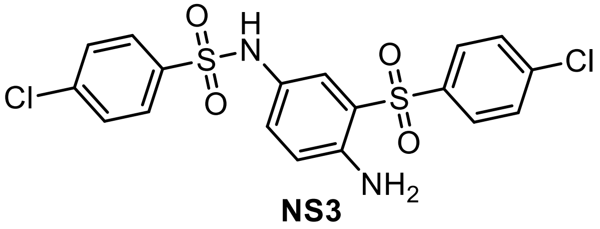

N-(4-amino-3-(4-chlorophenylsulfonyl)phenyl)-4-chlorobenzenesulfonamide

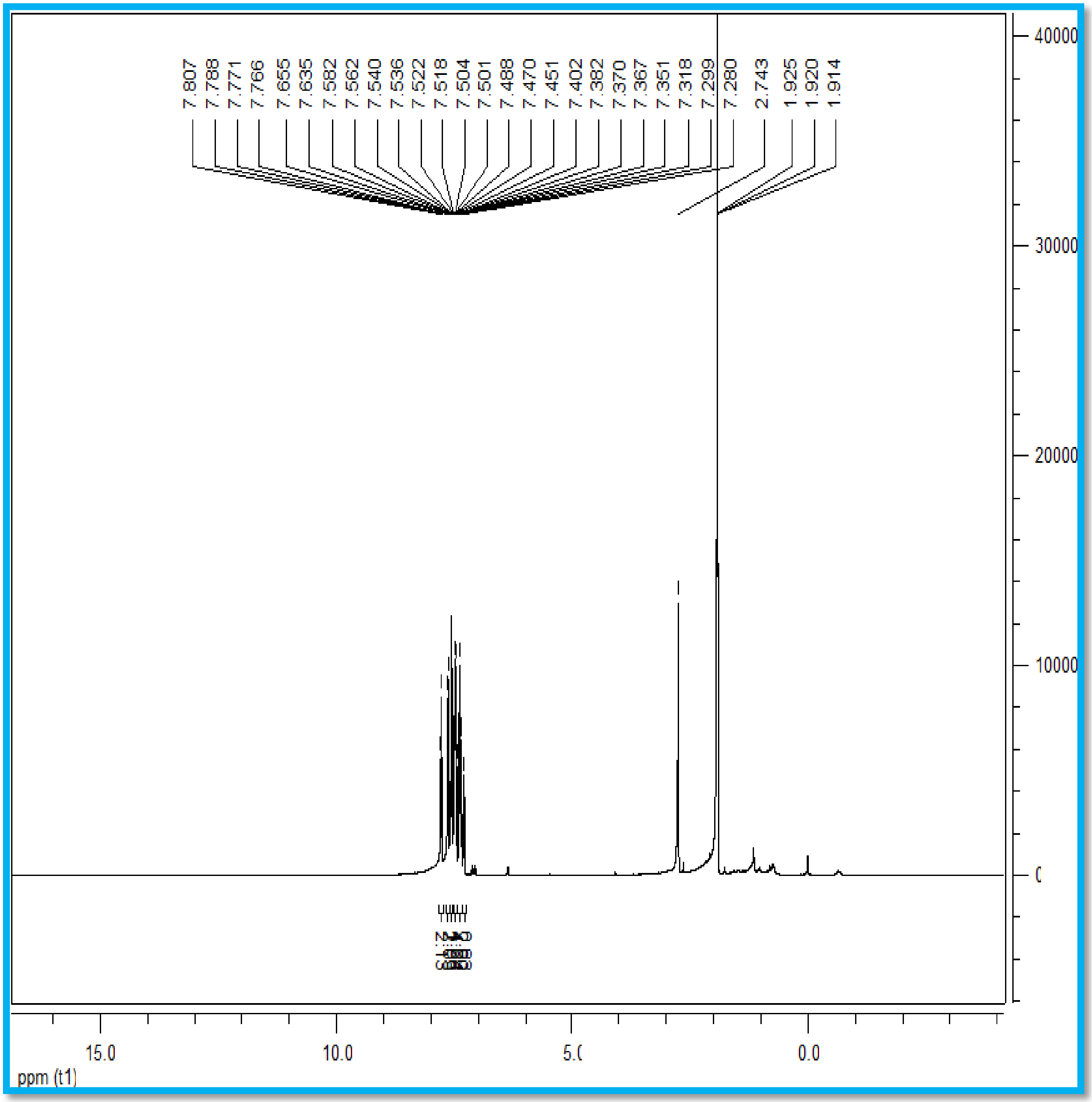

Expanded ^{1}H NMR spectrum of NS3

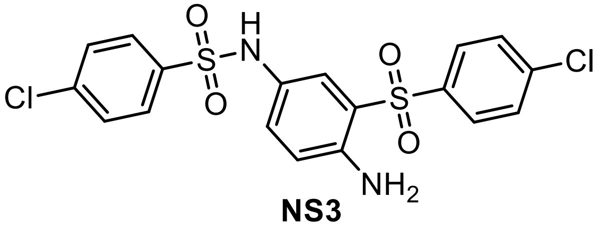

NS3

N-(4-amino-3-(4-chlorophenylsulfonyl)phenyl)-4-chlorobenzenesulfonamide

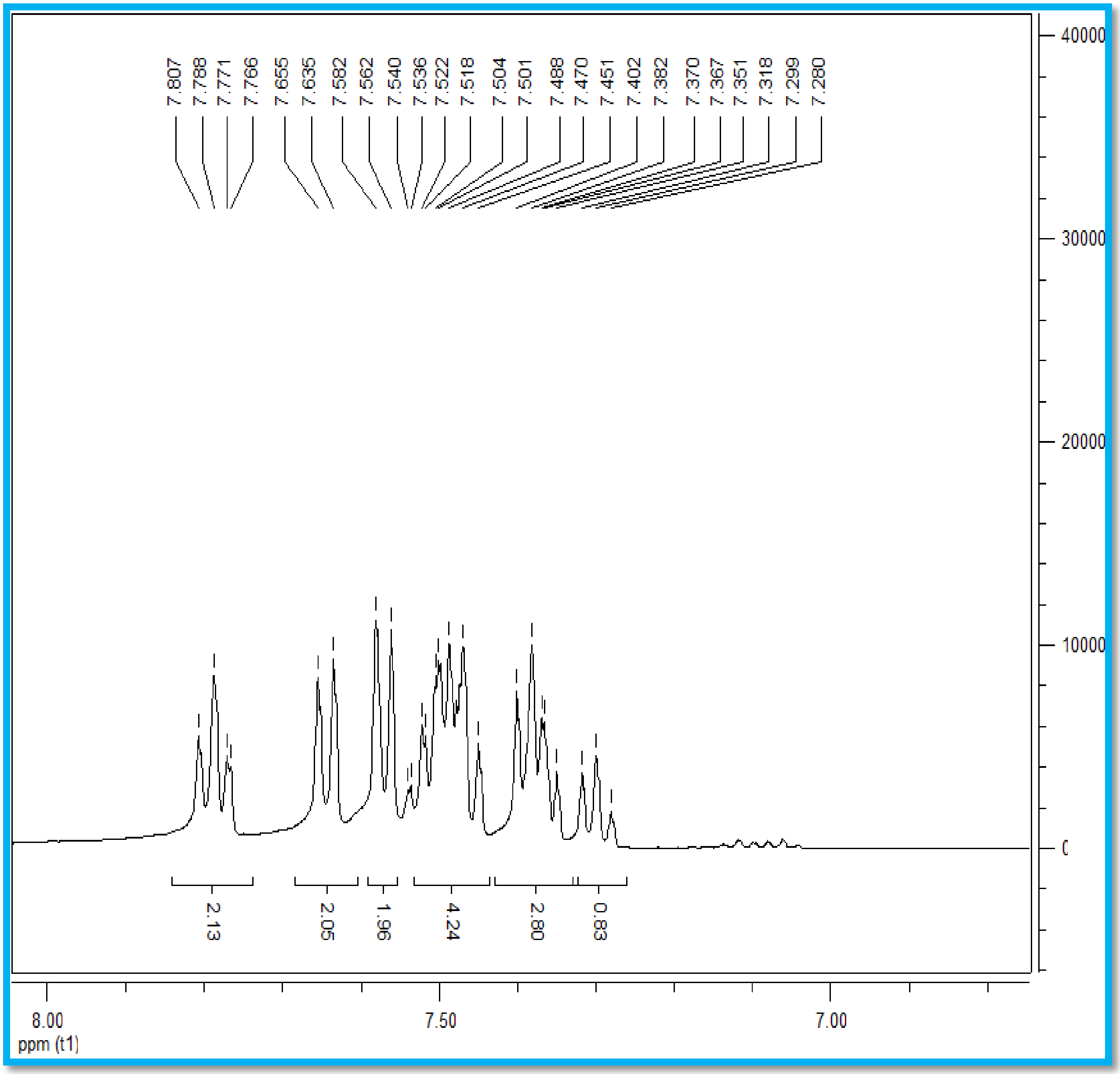

^{13}C NMR spectrum of NS3

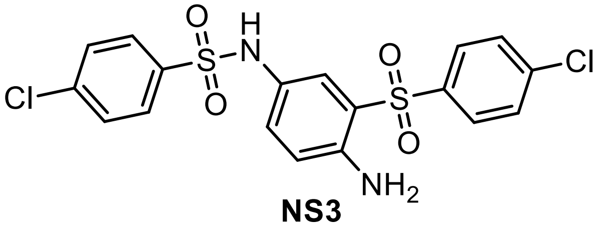

NS3

N-(4-amino-3-(4-chlorophenylsulfonyl)phenyl)-4-chlorobenzenesulfonamide

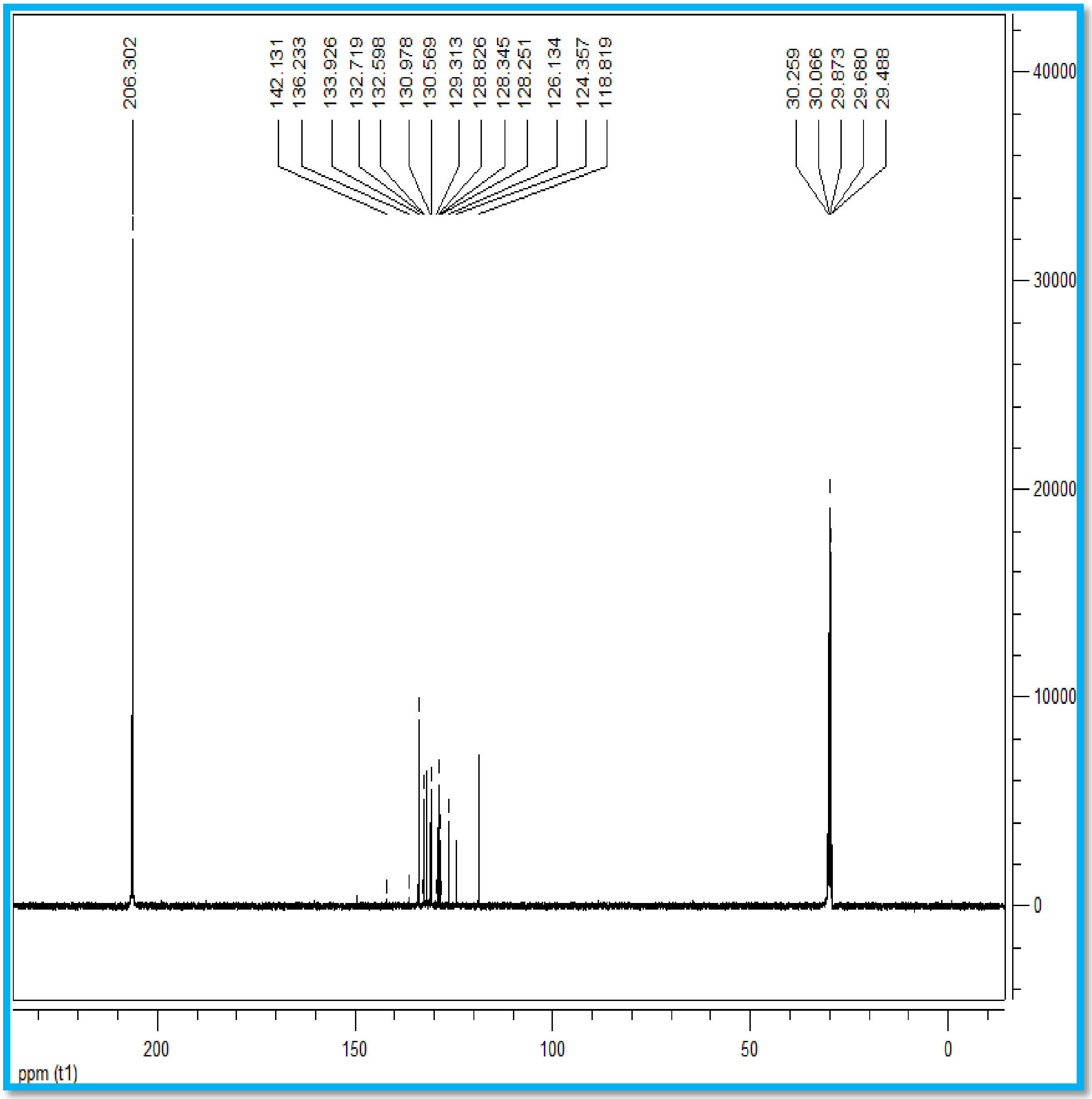

MS spectrum of NS3

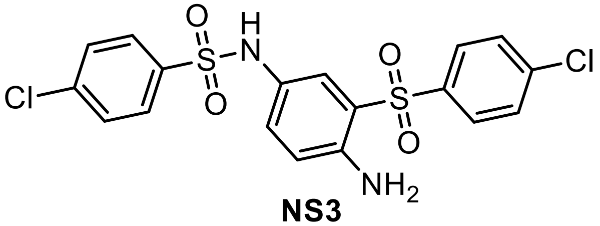

NS3

N-(4-amino-3-(4-chlorophenylsulfonyl)phenyl)-4-chlorobenzenesulfonamide

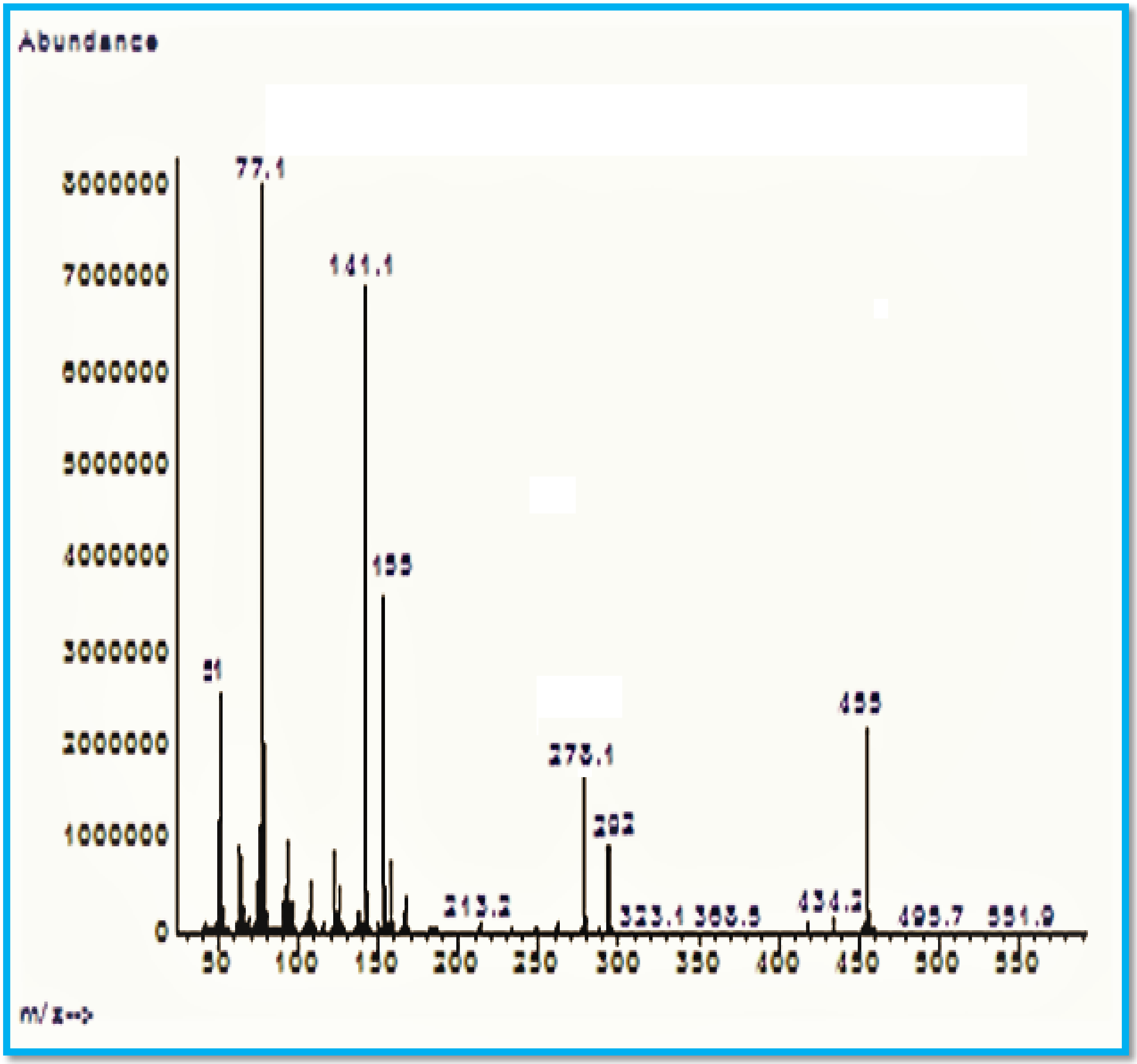

Supplement: Supplementary file 1 — Supporting Information [file 41598_2019_38544_MOESM1_ESM.pdf]
